# A Data-Driven Approach to Assessing Hepatitis B Mother-to-Child Transmission Risk Prediction Model: Machine Learning Perspective

**DOI:** 10.2196/69838

**Published:** 2025-05-23

**Authors:** Dung Nguyen Tien, Huong Thi Thu Bui, Tram Hoang Thi Ngoc, Thuy Thi Pham, Dac Trung Nguyen, Huyen Nguyen Thi Thu, Thi Thu Hang Vu, Thi Lan Anh Luong, Lan Thu Hoang, Ho Cam Tu, Nina Körber, Tanja Bauer, Lam Khanh Ho

**Affiliations:** 1 Department of Microbiology Thai Nguyen University of Medicine and Pharmacy Thái Nguyên Vietnam; 2 Department of Immunology - Molecular Genetics Thai Nguyen National General Hospital Thái Nguyên Vietnam; 3 Department of MBG Hanoi Medical University Hanoi Vietnam; 4 Center of Clinical Genetics and Genomics Hanoi Medical University Hospital Hanoi Vietnam; 5 Technical University of Munich Munich Germany; 6 Institute of Virology (VIRO) Molecular Targets and Therapeutics Center Helmholtz Zentrum München Munich Germany; 7 Faculty of Information Technology Hung Yen University of Technology and Education Hưng Yên Vietnam

**Keywords:** chronic hepatitis B virus infection, liver, pregnant women, cord blood, PBMCs (peripheral blood mononuclear cells), ID3 (Iterative Dichotomiser 3), CART (classification and regression trees)

## Abstract

**Background:**

Hepatitis B virus (HBV) can be transmitted from mother to child either through transplacental infection or via blood-to-blood contact during or immediately after delivery. Early and accurate risk assessments are essential for guiding clinical decisions and implementing effective preventive measures. Data mining techniques are powerful tools for identifying key predictors in medical diagnostics.

**Objective:**

This study aims to develop a robust predictive model for mother-to-child transmission (MTCT) of HBV using decision tree algorithms, specifically Iterative Dichotomiser 3 (ID3) and classification and regression trees (CART). The study identifies clinically and paraclinically relevant predictors, particularly hepatitis B e antigen (HBeAg) status and peripheral blood mononuclear cell (PBMC) concentration, for effective risk stratification and prevention. Additionally, we will assess the model’s reliability and generalizability through cross-validation with various training-test split ratios, aiming to enhance its applicability in clinical settings and inform improved preventive strategies against HBV MTCT.

**Methods:**

This study used decision tree algorithms—ID3 and CART—on a data set of 60 hepatitis B surface antigen (HBsAg)–positive pregnant women. Samples were collected either before or at the time of delivery, enabling the inclusion of patients who were undiagnosed or had limited access to treatment. We analyzed both clinical and paraclinical parameters, with a particular focus on HBeAg status and PBMC concentration. Additional biochemical markers were evaluated for their potential contributory or inhibitory effects on MTCT risk. The predictive models were validated using multiple training-test split ratios to ensure robustness and generalizability.

**Results:**

Our analysis showed that 20 out of 48 (based on a split ratio of 0.8 from a total of 60 cases, 42%) to 27 out of 57 (based on a split ratio of 0.95 from a total of 60 cases, 47%) training cases with HBeAg-positive status were associated with a significant risk of MTCT of HBV (χ^2^_8_=21.16, *P*=.007, *df*=8). Among HBeAg-negative women, those with PBMC concentrations ≥8 × 10^6^ cells/mL exhibited a low risk of MTCT, whereas individuals with PBMC concentrations <8 × 10^6^ cells/mL demonstrated a negligible risk. Across all training-test split ratios, the decision tree models consistently identified HBeAg status and PBMC concentration as the most influential predictors, underscoring their robustness and critical role in MTCT risk stratification.

**Conclusions:**

This study demonstrates that decision tree models are effective tools for stratifying the risk of MTCT of HBV by integrating key clinical and paraclinical markers. Among these, HBeAg status and PBMC concentration emerged as the most critical predictors. While the analysis focused on untreated patients, it provides a strong foundation for future investigations involving treated populations. These findings offer actionable insights to support the development of more targeted and effective HBV MTCT prevention strategies.

## Introduction

Hepatitis B virus (HBV) mother-to-child transmission (MTCT) can occur through transplacental infection or blood-to-blood contact during or after delivery and accounts for a significant proportion of chronic HBV infections worldwide [1]. In high-prevalence countries such as Vietnam, MTCT remains the most common mode of transmission. Children who acquire chronic infection have a 40% lifetime risk of dying from HBV-related complications. Antiviral treatment to reduce high viral loads, along with immunoprophylaxis using anti-HBV immunoglobulin shortly after delivery and active HBV immunization of neonates, can significantly reduce the incidence of MTCT. However, these measures are rarely implemented in most countries [2]. Studies examining high-risk factors associated with MTCT in patients with HBV lack depth, and the relationship between HBV DNA in maternal serum and cord blood remains unclear. We conducted a clustering study to explore the potential link between HBV infection in pregnant women and cord blood, aiming to identify a clinical reference marker for prenatal surveillance and postnatal management, and to strengthen the prevention of HBV MTCT.

Data mining techniques play a crucial role in clinical decision-making by providing physicians with accurate, reliable, and timely predictions through various models. Machine learning is broadly categorized into 3 types: supervised learning, unsupervised learning, and reinforcement learning. The decision tree is a supervised learning algorithm capable of handling both regression and classification tasks. A typical machine learning algorithm involves 2 main steps: training (where the algorithm learns a model from data) and prediction (where the learned model is used to predict new values). The training step in the decision tree algorithm constructs a decision tree. A decision tree is an effective support tool for engineers’ decision-making [3], using a tree model that illustrates decisions and their possible outcomes, including random outcomes, resource costs, and benefits. Based on published premise cluster analysis research, we set up a machine learning experiment. This research may help identify potential risk factors for MTCT.

This study was conducted at a single center in Thai Nguyen, Vietnam. It is valuable to explore how variations in HBV circulation rates, genotypes, and health care practices across different regions of the country might influence both the detection and relevance of the study. Thai Nguyen, where the research took place, serves as the economic, political, and social hub of the northeastern region, the Central Highlands, and the northern mountainous areas of Vietnam. As a result, although the patients come from various hometowns, they share characteristics typical of the northern mountainous region, where many ethnic minorities reside. Viral hepatitis—particularly HBV, hepatitis D virus, and hepatitis E virus—remains a significant public health concern in Vietnam, especially among ethnic minority communities. Higher infection rates in these groups are often linked to limited access to health care, low socioeconomic status, high-risk living conditions, and a lack of awareness about the disease.

In this study, we enrolled 60 pregnant women who tested positive for hepatitis B surface antigen (HBsAg) from a clinical setting, applying strict inclusion criteria to ensure that only those with chronic HBV infection—defined as being HBsAg-positive for more than 6 months—and who had not received any HBV treatment were included.

In our study, we focused exclusively on HBsAg-positive pregnant women who were not receiving antiviral treatment. This approach was intentionally chosen to better understand the natural history and intrinsic risk factors for MTCT in untreated patients. According to World Health Organization (WHO) guidelines and current Vietnamese recommendations, pregnant women identified as being at high risk of MTCT are typically advised to begin treatment early. However, because sampling in our study took place either before delivery or immediately at the time of delivery, some participants had not yet been diagnosed or had not had the opportunity to access treatment.

After delivery, we strongly recommended that all patients initiate antiviral therapy promptly, emphasizing that postnatal treatment does not negatively impact milk production or the quality of breast milk for the infant. Although excluding women who were already receiving treatment may limit the generalizability of our findings, our study offers valuable baseline data on MTCT risk in untreated patients. This baseline can serve as a crucial reference for future studies evaluating the effectiveness of early treatment interventions.

After obtaining informed consent, we collected maternal and cord blood samples to measure various biomarkers, including viral markers (HBsAg, hepatitis B e antigen [HBeAg], HBV DNA) and biochemical parameters (eg, alanine aminotransferase [ALT], aspartate aminotransferase [AST], and peripheral blood mononuclear cells [PBMCs]). The raw data were preprocessed using R (R Foundation), which involved cleaning and organizing the data for further analysis. We conducted descriptive and univariate analyses to summarize the characteristics of the study population, followed by correlation and clustering analyses to explore relationships among the measured variables. Based on logistic regression models and initial analyses, we identified specific cutoff values (eg, PBMCs/mL and HBV DNA copies/mL). Feature selection was performed using information gain techniques based on Iterative Dichotomiser 3 (ID3) theory, identifying key predictors such as HBeAg status and PBMC levels. The selected features were then used to construct decision tree models with both the ID3 and classification and regression trees (CART) algorithms to classify participants into distinct MTCT risk categories.

**Figure 1:**
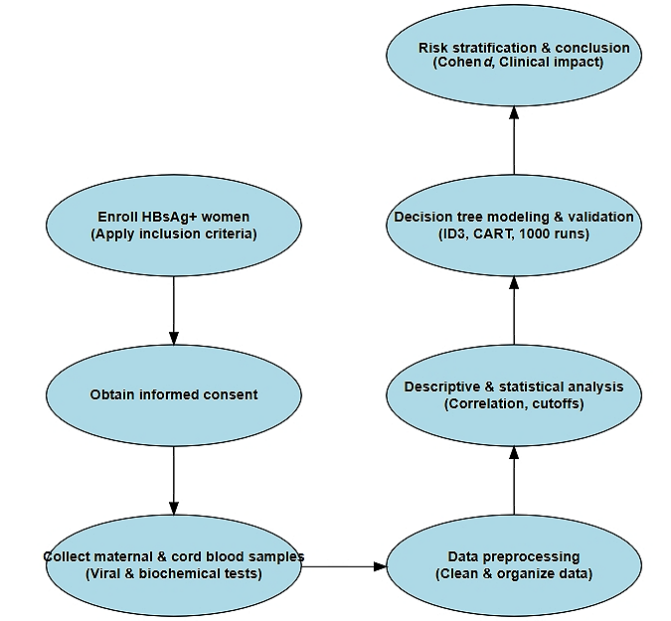
Study flowchart. CART: classification and regression trees; HBsAg: hepatitis B surface antigen; ID3: Iterative Dichotomiser 3.

We validated the models through 1000 simulation runs using various training-test split ratios, calculating performance metrics—including accuracy, sensitivity, specificity, and AUC—to assess model performance. The validated models enabled risk stratification based on Cohen effect size classifications (trivial, small, medium, and large), and the results were interpreted to provide actionable clinical insights, highlighting the critical roles of HBeAg and PBMCs in predicting the risk of HBV MTCT (Figure 1).

The goal of this study is to develop and validate a machine learning–based decision tree model to effectively predict the risk of HBV transmission from mother to child. This will be accomplished by incorporating key clinical and paraclinical markers—particularly HBeAg status and PBMC concentration—to inform targeted prevention strategies.

## Methods

### Study Population

Between 2020 and 2021, we conducted a pilot study at Thai Nguyen National Hospital in Vietnam, focusing on pregnant women who received regular check-ups and delivered at the facility. A total of 60 pregnant women who tested positive for HBsAg were enrolled.

Participants were provided with detailed information about the risks of hepatitis B transmission and were counseled on available treatment options. They could choose to begin treatment immediately or defer it until after delivery. Following birth, both mothers and their newborns were monitored for 3-6 months. Newborns received routine prophylactic care, and mothers with HBV DNA levels exceeding 200,000 IU/mL were offered treatment in accordance with WHO guidelines (2021) [4].

Only pregnant women with chronic hepatitis B (CHB) infection (HBsAg positive for more than 6 months) who opted to delay antiviral treatment were included in the study. We collected various biological samples and conducted clinical surveillance to establish a comprehensive hepatitis B patient cohort, aimed at exploring the multifactorial risk factors associated with HBV MTCT.

### Ethical Considerations

This study was approved by the Institutional Review Board for Ethics in Biomedical Research of Hanoi Medical University (approval number NCS22/HMU-IRD), ensuring the protection of participants’ rights. Informed consent for future data use was obtained during the original data collection, and this secondary analysis was conducted in accordance with that provision. All data used in this study were deidentified and securely stored. No personally identifiable information was collected or retained, and no identifiable images of individuals are included in the manuscript or multimedia appendices.

### Diagnostic Criteria

We collected clinical data from pregnant women who tested positive for HBsAg and were not receiving HBV treatment. The information gathered included general demographic and obstetric details such as age at delivery, gestational age, and pregnancy history (including the number of pregnancies and births, cesarean sections, and any preexisting conditions). Laboratory test results—such as ALT, AST, HBV DNA levels, and records of any antiviral therapy—were also included. Additionally, we assessed various maternal conditions, including preeclampsia, chronic hypertension, history of abortion, placental abruption, hyperthyroidism, gestational diabetes mellitus, pregnancy-induced hypertension, intrahepatic cholestasis of pregnancy, and other related complications. A retrospective analysis was conducted to examine the association between HBV infection status and preclinical factors.

### Isolation of Peripheral Blood Mononuclear Cells

We collected maternal blood in EDTA (ethylenediaminetetraacetic acid) tubes and also collected umbilical cord blood. The cord blood was obtained from the umbilical vein of the umbilical cord and placed in a 20-mL cylinder with anticoagulant immediately after birth. The process of collecting cord blood takes only 2-3 minutes and involves the following steps: (1) Immediately after the mother gives birth, the medical staff clamps a section of the umbilical cord that is at least 10 cm long. (2) This section can be cut immediately to obtain a blood sample or left intact until after the placenta is delivered. (3) The surface of the umbilical cord is disinfected with povidone-iodine solution. (4) The needle of the collection cylinder is then inserted into the umbilical vein to draw the blood. (5) The collection cylinder is clamped, and the needle is withdrawn. (6) Finally, the collection cylinder is gently shaken to mix the blood with the anticoagulant.

Serum and plasma samples were analyzed for viral markers (HBsAg, HBeAg, and HBV DNA copies/mL) and other preclinical factors, including platelet count (×10^3^ cells/mL), prothrombin time (seconds), prothrombin ratio (%), hemoglobin (g/L), red blood cell count (×10^6^ cells/mL), creatinine (µmol/L), AST (U/L), and ALT (U/L) [5].

### Statistical and Decision Tree Analysis

Data collection, storage, and analysis in this study were conducted using the R 4.1.0 package tools. The correlation *R* value measures the strength of the linear relationship between 2 quantitative variables. The Pearson *R* formula is as follows:



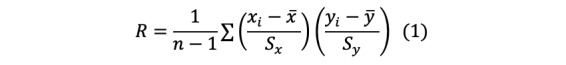



where *R* is the Pearson correlation coefficient; and *x* and *y* are 2 vectors of length *i* and *j*, respectively [6]. The value of *R* ranges from –1 to 1, with *R*>0 indicating a positive association and *R*<0 indicating a negative association [6].

We conducted the clustering analysis again using the 5 most significant factors identified in our earlier study [7-9]. To determine the effect size for each factor, we transformed the natural logarithm of the odds ratio, that is, ln(odds ratio), by dividing it by 1.81, based on their respective odds ratios [10].

Additionally, we utilized the ID3 algorithm to generate general rules and predictions for new cases. This algorithm requires specifying the order in which attributes are evaluated at each step. As finding the optimal solution can be challenging when dealing with numerous attributes (such as varied patient test results), we opted for a simpler approach: at each step, we selected the attribute that best satisfied a chosen criterion. After selecting an attribute, the data are divided into child nodes based on their values, and this process continues recursively for each child node. Although this greedy selection method may not always yield the optimal solution, it is intuitively close to the best outcome and significantly simplifies the problem [11].

A crucial element of this approach is assessing the quality of each partition. Ideally, a good partition is one in which each child node predominantly contains data from a single class, allowing it to be treated as a leaf with no further division. Conversely, a partition that produces child nodes with mixed classes is less desirable. To evaluate this, we require a function that measures the purity or impurity of a partition. This function should yield the lowest value when each node contains data from only 1 class (indicating high purity), and a higher value when nodes include a diverse mix of classes. The entropy function, commonly used in information theory, serves this purpose (see equation 2).



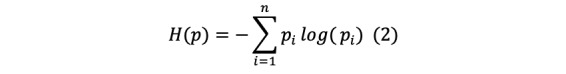



In the ID3 algorithm, the loss function for a decision tree is defined as the weighted sum of the entropies at its leaf nodes, with the weights corresponding to the number of data points in each node. The goal of ID3 is to determine the order of attribute splits in a way that minimizes this total loss. This is achieved by selecting the attribute that leads to the greatest reduction in entropy at each step. Essentially, constructing the decision tree using ID3 can be viewed as a series of smaller tasks, where at each nonleaf node, we choose the attribute that most effectively improves the split. We then develop a calculation method for each of these nodes (see equation 3). Thus, the entropy at this node is given by:



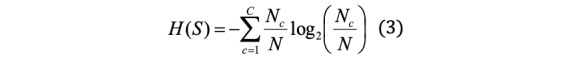



Next, suppose the selected attribute is *x*. We define it as the weighted sum of the entropy of each *child node*, computed similarly to equation 3. This weighting is important because *nodes often* contain different numbers of points (see equation 4).



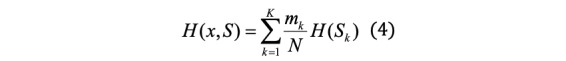



Let the selected attribute be *x*. Based on *x*, the data points in *S* are divided into *K* *childnode S*_1_, *S*_2_, ..., *S*_K,_ with the number of points in each *child node* being *m*_1_, *m*_2_, ..., *m*_K_, respectively.

Next, we define the *information gain* based on the attribute *x*, as given by equation 5.

*G*(*x*, *S*) = *H*(*S*) – *H*(*x*,*S*) **(5)**

Let the selected attribute be *x*. Based on *x*, the data points in *S* are divided into *K* *childnode S*_1_, *S*_2_, ..., *S*_K_, with the number of points in each *child node* being *m*_1_, *m*_2_, ..., *m*_K_, respectively. In equation 5, *H*(*s*) is the *root node entropy.* In ID3, at each node, the selected attribute is determined based on equation 6, which identifies the property that maximizes the information gain.



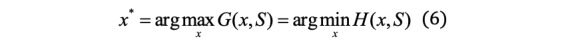



Identifying important variables helps eliminate less important ones, simplifying the model and reducing noise. Thus, the final step determines the importance of each variable using a different splitting ratio. These variables can be quantified by the reduction in impurity (such as the Gini index) achieved when they are used for splitting. This imputation follows the CART formula (see equations 7 and 8).



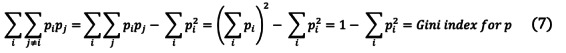



Let us suppose an object is selected at random from one of the C classes according to the probabilities (*p*_1_, *p*_2_, ..., *p*_C_) and is randomly assigned to a class using the same distribution. In this scenario, we get the following:



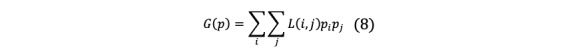



In equation 8, let *L*(*i, j*) be the loss of assigning class *j* to an object which actually belongs to class *i.* The expected cost of misclassification is 
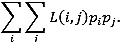


In our study, we simulate 1000 runs for each splitting ratio. The best variable is the one that shows the highest Gini index score. The Gini index has been adapted to assess health inequality across populations by providing estimates that capture the distribution of risk, or lack of risk, among the entire population or within specific groups [12].

## Results

### Power Estimation in a Multivariate Regression

**Figure 2:**
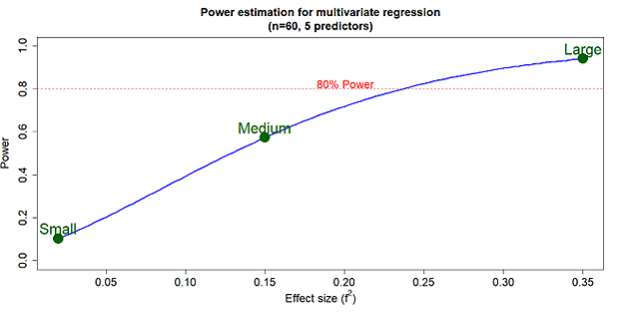
Power estimation in a multivariate regression with 60 participants and 5 predictors. We set n=60 (sample size) and *p*=5 (number of predictors). The error degrees of freedom are calculated as *v*=60 − 5 − 1 = 54. A sequence of *f*^2^ values ranging from 0.02 (small) to 0.35 (large) is generated. For each *f*^2^ value, we use pwr.f2.test() to estimate the power of the overall F-test in a multiple regression. The y-axis shows statistical power, and the x-axis shows the effect size (*f*^2^). A horizontal red dashed line marks the 80% power threshold. Conventional effect sizes (small, medium, and large) are highlighted with dark green points and labeled accordingly.

We generate a power estimation curve for a multivariate regression (using *f*^2^ as the effect size measure) with a total sample size of 60 and 5 predictors. This plot helps illustrate how adequate—or inadequate—the sample size is for detecting effects in a multivariate context. In multiple regression, Cohen [13] suggested the following guidelines for *f*^2^ effect sizes: small (*f*^2^=0.002), medium (*f*^2^=0.15), and large (*f*^2^=0.35). We calculate the statistical power for a range of *f*^2^ values given the following: total sample size N=60, number of predictors *p*=5, degrees of freedom for the error *v*=*N*–*p*–1=54, and significance level α=.05. Figure 2 illustrates how statistical power changes in a multiple regression model (with 5 predictors and a total sample size of 60) as the effect size (*f*^2^) ranges from small (0.02) to large (0.35). The vertical axis shows the probability (power) of detecting an actual effect at the 5% significance level, while the horizontal axis shows the size of that effect. The red dashed line at 0.80 marks the conventional threshold for sufficient power (80%). When (*f*^2^) is small (0.02), the power is around 0.2, indicating only a 20% chance of detecting such a minor effect with the given sample size and number of predictors. As (*f*^2^) approaches medium (0.15), the power increases but remains below the 0.80 line, suggesting that moderate effects are not reliably detected. Only when (*f*^2^) nears the large range (around 0.35) does the power surpass or approach the 80% mark, implying that the study can reliably detect larger effects but may struggle to identify smaller or moderate ones (Figure 2).

### The Five Most Important Factors of MTCT Risk: HBeAg, ALT, AST, HBV DNA, and PBMCs

The mean age is 27.6 (SD 4.2) years. Pregnancy for the second time or more accounted for 43 out of 60 (72%) cases. Clinical symptoms were edema, fatigue, and loss of appetite, similar to those experienced during pregnancy; 40 (67%) women had cesarean section. Of the 60 women, 27 (45%) were HBeAg positive, and HBV DNA ≥ 10^7^ copies/mL was reported in 20 (33%). The mean gestational age is 38.9 (SD 1.2) weeks. Among the babies, 32 (53%) were HBsAg positive, and 23 (38%) were HBeAg positive (Tables 1-4).

In 60 Vietnamese CHB pregnant women, 32 (53%) cord-blood samples were HBsAg positive, and 28 (47%) were HBsAg negative. We fit a logistic regression model to predict the subclinical values (cutoff value), which correspond to 50:50 probabilities that HBsAg in cord blood is positive. Two variables have a positive association when above-average values of one tend to accompany above-average values of the other, and below-average values tend to occur together as well. Two variables have a negative association when above-average values of one tend to accompany below-average values of the other [14] (see Table 5 and Figure S1 in Multimedia Appendix 1).

**Table 1:** General characteristics of 60 Vietnamese pregnant women with chronic hepatitis B^a^.

General characteristics of the study participants		Values, n (%)	
**Mother’s age (years)**			
	18-35	58 (97)	
	>35	2 (3)	
	Mean (SD)	27.6 (4.2)	
**Number of pregnancies**			
	First time	17 (28)	
	From the second time	43 (72)	
**Time of detection of hepatitis B virus infection**			
	Before getting pregnant	25 (42)	
	This time	35 (58)	

^a^32 (53%) cord blood samples were hepatitis B surface antigen positive, and 28 (47%) hepatitis B surface antigen negative.

**Table 2:** Clinical and preclinical characteristics of the 60 Vietnamese pregnant women with chronic hepatitis B.

Characteristics			Values, n (%)
**Clinical characteristics**			
	Edema		7 (12)
	Tired		8 (13)
	Anorexia		1 (2)
	Nausea/vomiting		4 (7)
	Insomnia		4 (7)
	Joint pain		3 (5)
	Right lower quadrant pain		2 (3)
	**Birth method**		
		Birth without episiotomy	4 (7)
		Birth with episiotomy	16 (27)
		Caesarean section	40 (67)
**Preclinical characteristics**			
	An increase of aspartate aminotransferase		14 (23)
	An increase of alanine aminotransferase		11 (18)
	Hepatitis B e antigen positive		27 (45)
	Hepatitis B e antigen negative		33 (55)
	Hepatitis B virus DNA≥10^7^		20 (33)
	Hepatitis B virus DNA<10^7^		40 (67)

**Table 3:** Clinical and preclinical characteristics of babies (N=60).

Characteristics			Values
**Clinical**			
	Apgar score≥8, n (%)		58 (97)
	Apgar score<7, n (%)		2 (3)
	Gestational age, mean (SD)		38.9 (1.2)
	**Weight (g)**		
		<3500, n (%)	47 (78)
		≥3500, n (%)	13 (22)
		Mean (SD)	3198.3 (362.9)
**Preclinical (cord blood), n (%)**			
	Hepatitis B surface antigen positive		32 (53)
	Hepatitis B surface antigen negative		28 (47)
	Hepatitis B e antigen positive		23 (38)
	Hepatitis B e antigen negative		37 (62)

**Table 4:** Preclinical measures.

Details of preclinical measures results	Range	Median (95% CI)	Mean (SD)
Maternal prothrombin time (seconds) (N=60)	10.5-47.8	12.1 (11.6-12.8)	12.9 (4.76)
Maternal prothrombin ratio (%) (N=60)	10.0-133.0	108.0 (99.6-117.0)	106 (21.6)
Maternal red blood cells (×10^6^ cells/mL) (N=60)	3.25-6.25	4.33 (4.06-4.69)	4.38 (0.539)
Maternal platelet count (×10^3^ cells/mL) (N=60)	109.0-344.0	214.0 (180.0-260.0)	219 (59.6)
Maternal creatinine (µmol/L) (N=60)	41.0-99.0	59.6 (54.4-68.8)	62.1 (11.1)
Maternal aspartate transaminase (U/L) (N=60)	13.0-285.0	22.1 (18.6-29.2)	33.2 (41.3)
Maternal alanine transaminase (U/L) (N=60)	6.40-217.0	15.8 (12.8-24.3)	27.7 (38.0)
Maternal protein in blood (g/L)	12.3-80.2	67.9 (65.8-71.1)	66.7 (9.82)
Maternal albumin in blood (g/L)	25.6-44.9	34.5 (33.0-35.7)	34.4 (3.54)
Maternal hepatitis B virus DNA (copies/mL) (N=60)	35.0-1,350,000,000	49,600 (771.0-129,000,000)	152,000,000 (310,000,000)
Maternal peripheral blood mononuclear cells (cells/mL) (N=60)	1,300,000-12,300,000	5,500,000 (3,000,000-7,510,000)	5,620,000 (3,050,000)
Cord blood mononuclear cell concentration (cells/mL) (N=60)	3,640,000-51,000,000	12,200,000 (6,500,000-15,000,000)	12,600,000 (7,630,000)

**Table 5:** Cutoff values.

Variables	Cutoff_50_^a^ for hepatitis B surface antigen–positive probability in cord blood	The direction of the relationship between the 2 variables	Cross-reference
Maternal peripheral blood mononuclear cell concentration	8.03 × 10^6^ cells/mL	Negative	Figure S1A in Multimedia Appendix 1
Maternal hepatitis B virus DNA	5.40 × 10^7^ copies/mL	Positive	Figure S1B in Multimedia Appendix 1
Maternal platelet count	317.89 × 10^3^ cells/mL	Negative	Figure S1C in Multimedia Appendix 1
Maternal prothrombin time	11.00 seconds	Positive	Figure S1D in Multimedia Appendix 1
Cord blood mononuclear cell concentration	6.64 × 10^6^ cells/mL	Positive	Figure S1E in Multimedia Appendix 1
Maternal hemoglobulin	128.53 g/L	Negative	Figure S1F in Multimedia Appendix 1
Maternal red blood cells	5 × 10^6^ cells/mL	Negative	Figure S1G in Multimedia Appendix 1
Maternal creatinine	37.46 µmol/L	Positive	Figure S1H in Multimedia Appendix 1
Maternal aspartate aminotransferase	14.15 U/L	Positive	Figure S1I in Multimedia Appendix 1
Maternal alanine aminotransferase	43.34 U/L	Negative	Figure S1K in Multimedia Appendix 1
Maternal prothrombin ratio	76.34%	Positive	Figure S1L in Multimedia Appendix 1

^a^Predicted values linked with a 50:50 probability that hepatitis B surface antigen is detectable in cord blood.

**Figure 3:**
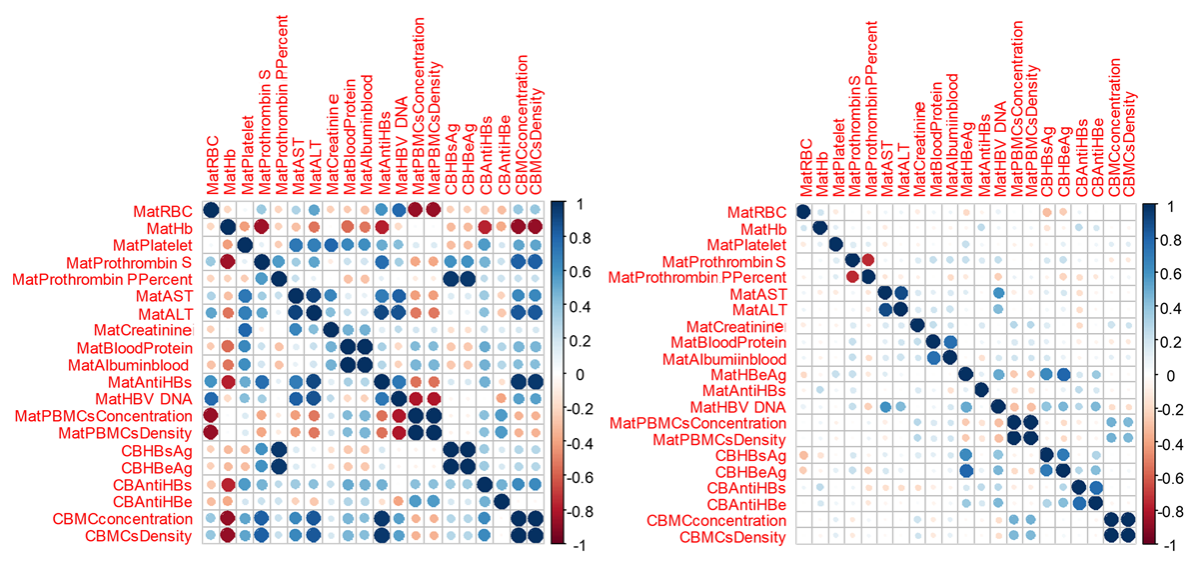
Correlation matrix between biomarkers depicted as a heat map. The heat map compares 2 groups: HBV DNA ≥ 5 × 10⁷ copies/mL (left panel) and HBV DNA < 5 × 10⁷ copies/mL (right panel). It illustrates the color-coded Pearson correlation coefficients between subclinical indices, including prothrombin time, AST, ALT, RBC, and haemoglobin in maternal blood; the concentration and density of PBMCs; and the status of HBeAg and anti-HBs in both cord and maternal blood. Cell colors represent the strength and direction of correlations, ranging from red (negative correlation) to blue (positive correlation). The intensity of the color reflects the magnitude of the correlation, as indicated by the color scale shown to the right of the panel. Pairwise Pearson correlation coefficients are detailed in Multimedia Appendices 2-5. ALT: alanine aminotransferase; AST: aspartate aminotransferase; CB: cord blood; CBMC: cord blood mononuclear cell; Hb: hemoglobin; HBeAg: hepatitis B e antigen; HBsAg: hepatitis B surface antigen; RBC: red blood cell.

We study the Pearson correlation between each factor in the matrix model. The *R* score with a significant *P* value will be considered for further steps (see Figure 3 and Multimedia Appendices 2-5). When maternal viral load exceeds 5 × 10^7^ copies/mL, the risk of being HBsAg positive in cord blood increases by 123% (risk ratio 2.23, 95% CI 1.48-3.36); when the viral load is lower than this baseline, the risk decreases by 55% (risk ratio 0.45, 95% CI 0.30-0.67; *P*<.001; see Table 6 and Multimedia Appendices 6 and 7). We calculate the risk ratio and odds ratio based on the new value indications (see Table 6) for 2 groups: HBsAg cord blood positive and negative, using the results of the HCA analysis. A dendrogram and principal component analysis plot were constructed based on the correlation between each factor (see Multimedia Appendices 8-10).

**Table 6:** Risk ratios for hepatitis B surface antigen–positive cord blood by factor, with corresponding threshold values.

Variables		Cord blood hepatitis B surface antigen positive (n=32), n	Cord blood hepatitis B surface antigen negative (n=28), n	Risk ratio (95% CI)	OR (95% CI)	Chi-square (*df*)	*P* value>chisq
**Maternal peripheral blood mononuclear cell concentration (cells/mL)**						0.34 (1)	.56
	≥8.03 × 10^6^	6	7	0.83 (0.44-1.58)	0.69 (0.20-2.38)		
	<8.06 × 10^6^	26	21	1.2 (0.63-2.28)	1.44 (0.42-4.96)		
**Maternal hepatitis B virus DNA (copies/mL)**						11.61 (1)	<.001
	≥5 × 10^7^	15	2	2.23 (1.48-3.36)	11.47 (2.32-56.65)		
	<5 × 10^7^	17	26	0.45 (0.30-0.67)	0.09 (0.02-0.43)		
**Maternal platelets (×10^3^cells/mL)**						0 (1)	>.99
	≥317.89	2	2	0.93 (0.34-2.56)	0.87 (0.11-6.59)		
	<317.89	30	26	1.07 (0.39-2.94)	1.15 (0.15-8.78)		
**Maternal prothrombin (seconds)**						0 (1)	>.99
	≥11	29	25	1.07 (0.46-2.48)	1.15 (0.21-6.27)		
	<11	3	3	0.93 (0.40-2.15)	0.86 (0.16-4.66)		
**Cord blood mononuclear cell concentration (cells/mL)**						0.73 (1)	.39
	≥6.64 × 10^6^	22	22	0.8 (0.49-1.29)	0.6 (0.19-1.94)		
	<6.64 × 10^6^	10	6	1.25 (0.77-2.02)	1.67 (0.52-5.38)		
**Maternal hemoglobin (g/L)**						0.46 (1)	.52
	≥128.53	11	12	0.84 (0.51-1.40)	0.7 (0.25-1.99)		
	<128.53	21	16	1.19 (0.71-1.98)	1.43 (0.50-4.07)		
**Maternal red blood cells (cells/mL)**						0.024 (1)	.88
	≥5 × 10^6^	2	3	0.73 (0.24-2.20)	0.56 (0.09-3.59)		
	<5 × 10^6^	30	25	1.36 (0.45-4.10)	1.8 (0.28-11.64)		
**Maternal creatinine (µmol/L)**						0.047 (1)	.83
	≥37.46	28	23	1.24 (0.57-2.67)	1.52 (0.37-6.33)		
	<37.46	4	5	0.81 (0.37-1.75)	0.66 (0.16-2.73)		
**Maternal aspartate transaminase (U/L)**						0.34 (1)	.56
	≥14.15	29	23	1.49 (0.59-3.76)	2.1 (0.45-9.73)		
	<14.15	3	5	0.67 (0.27-1.70)	0.48 (0.10-2.20)		
**Maternal alanine transaminase (U/L)**						0.61 (1)	.44
	≥43.34	4	1	1.57 (0.94-2.62)	3.86 (0.40-36.75)		
	<43.34	28	27	0.64 (0.38-1.06)	0.26 (0.03-2.47)		
**Maternal prothrombin ratio (%)**						0 (1)	>.99
	≥76.34	31	27	1.07 (0.26-4.36)	1.15 (0.07-19.25)		
	<76.34	1	1	0.94 (0.23-3.82)	0.87 (0.05-14.60)		

### PBMCs Gain the Most Information Following ID3 Theory Calculation

We next calculated the information gain for each factor in our actual data based on the correlation and clustering study results, following the ID3 theory. There are 5 attributes of pregnant women that may increase the risk of infection in infants. Each factor has 2 types of variants: HBeAg (positive and negative); ALT (<43.34 U/L and ≥43.34 U/L); AST (≥14.15 U/L and <14.15 U/L); HBV DNA (≥5 × 10^7^ and <5 × 10^7^ copies/mL); and PBMCs (≥8 × 10^6^ and <8 × 10^6^ cells/mL).

The analysis of Cohen *h* across the groups reveals varying degrees of effect size between the proportions of cord blood HBsAg-positive and -negative cases. Group 14 demonstrates a very large effect (*h*=1.46), characterized by a notably low proportion of positive cases (3/18, 17%) and a high proportion of negative cases (15/18, 83%). Group 12 shows a medium effect (*h*=0.51), indicating a moderate difference between the 2 proportions. Groups 10 (*h*=1.70) and 7 (*h*=1.85) both reflect very large effects, with high positive rates of 7 out of 8 (88%) and 9 out of 10 (90%), respectively, contrasted with much lower negative rates. Group 13 exhibits the maximum possible difference (*h*=3.14), with a proportion of 3 out of 3 (100%) positives and 0 out of 3 (0%) negatives, corresponding to the theoretical limit of Cohen *h*, π. By contrast, other_group shows a small effect (*h*=0.15), with nearly equal positive and negative proportions, suggesting minimal difference between the 2 groups (Multimedia Appendix 11). Using the pooled SD method, we calculated Cohen *d* to measure the standardized difference between groups 10 and 7 (Multimedia Appendix 11). For instance, if our calculated Cohen *d* is approximately 0.8 or higher, it suggests a large effect size, indicating that the difference in risk or related measures between these 2 groups is not only statistically significant but also clinically meaningful (*P*=.01 for group 10 and *P*=.001 for group 7).

In the 17 output values in Tables 1-4 (N=17 scores), there are 12 values showing effect increases: 1 value is small, 2 values are large (*P*=.01 for group 10 and *P*=.001 for group 7; also see Multimedia Appendix 11), and 2 are trivial. The probability that each data point falls into class c=*medium* is determined by *N*c*/N*=12/17. The probability that each data point falls into class c=*small* is determined by *N*c*/N*=1/17. The probability that each data point falls into class c=*large* is determined by *N*c*/N*=2/17, and the probability that each data point falls into class c=*trivial* is determined by *N*c*/N*=2/17. Therefore, the entropy at *the root node* is calculated according to equations 1 and 2 of this formula, with 2 classes “no” and “yes” (C=4), which is given as follows:



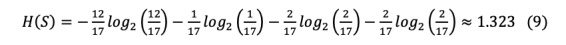



If one of the attributes—HBeAg, ALT, AST, HBV DNA, or PBMCs—is selected to divide the data, we calculate the weighted sum of the entropy of the child nodes. The result is shown in Table 5. PBMC concentrations were chosen because they have the highest information gain of 0.247. We could construct a decision tree by selecting PBMCs as the root node, as they provide the greatest information gain. From the root node (PBMCs), we branch out to other nodes, each named after the corresponding attribute (Multimedia Appendix 12).

### The Five Most Important Factors of MTCT Risk: HBeAg, ALT, AST, Serum HBV DNA, and PBMCs

**Figure 4:**
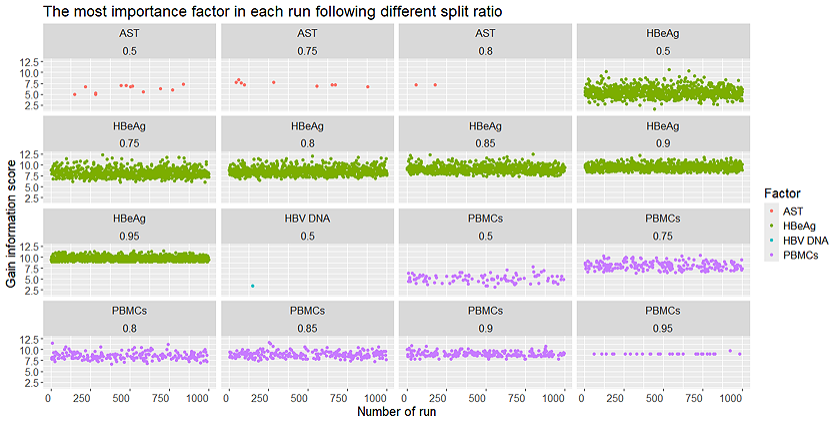
The dot plot of the most important factor in each run following different split ratios. AST: aspartate aminotransferase; HBeAg: hepatitis B e antigen; HBV: hepatitis B virus; PBMC: peripheral blood mononuclear cell.

We verified our calculation in R with 1000 runs (observations) for 5 split ratios: 0.50, 0.75, 0.80, 0.85, 0.90, and 0.95. The value in each node represents the number of observations in the data set that fall into that particular node. In Figure 4, we observed that the strongest information gain scores are found in HBeAg and PBMC concentration. From this dot plot, we can observe multiple runs of the ID3-based decision tree analysis at various training-test split ratios (shown in each column). In each run, the algorithm identifies the factor (AST, HBeAg, HBV DNA, or PBMCs) that provides the highest information gain for classification. The y-axis represents the extent of predictive “value” that each factor contributes. The results indicate that HBeAg (green dots) and PBMCs (purple dots) frequently exhibit high information gain, suggesting that these 2 factors play a prominent role in most runs, regardless of the split ratio. Meanwhile, AST (red dots) occasionally shows considerable information gain but appears less frequently, and HBV DNA (blue dot) rarely emerges as the most important factor. Overall, these findings highlight that HBeAg and PBMCs are the dominant factors in the model, maintaining stability across different training-test splits (see Figure 4).

**Figure 5:**
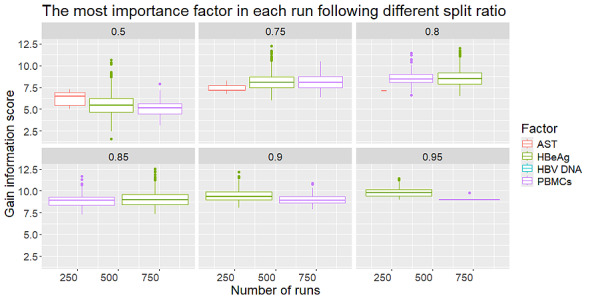
The bar plot of the most important factor in each run following different split ratios. AST: aspartate aminotransferase; HBeAg: hepatitis B e antigen; HBV: hepatitis B virus; PBMC: peripheral blood mononuclear cell.

Comparing the medians of the score values for HBeAg and PBMCs, we see that they are higher than those of the other factors. These dot plots show the information gain values for 4 factors (AST, HBeAg, HBV DNA, and PBMCs) across various runs of the model at different data split ratios (0.5, 0.75, 0.8, 0.85, 0.9, and 0.95). Each panel represents a specific split ratio, with the y-axis displaying the distribution of information gain scores for the factor deemed most important in that particular run. In general, HBeAg (green box) and PBMCs (purple box) consistently show higher median values or broader ranges of information gain, indicating that these 2 factors are crucial in the classification model, regardless of the training-test split used. In some panels (for instance, at split ratios of 0.5 or 0.75), AST (red box) occasionally records a high information gain score, but it does not match the stability or frequency of HBeAg or PBMCs. By contrast, HBV DNA (blue) seldom ranks with the highest information gain score (and hence not visible), suggesting it plays a lesser role in the classification decisions during these ID3 runs. Therefore, HBeAg and PBMCs are the primary contributors to the model’s predictive performance, while AST shows occasional dominance, and HBV DNA rarely serves as the top predictor (see Figure 5).

**Figure 6:**
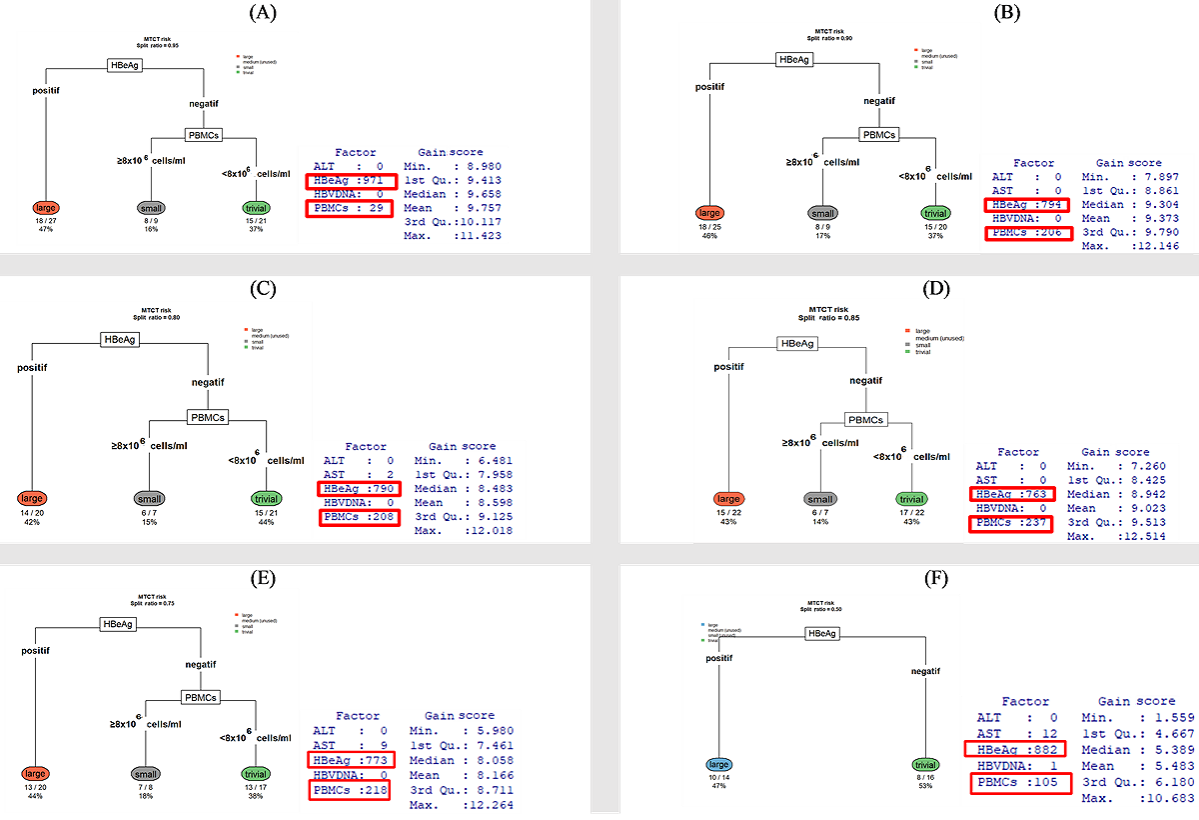
Decision tree diagram based on different split ratios: (A) 0.95, (B) 0.90, (C) 0.85, (D) 0.80, (E) 0.75, and (F) 0.50. Cohen classified effect sizes on MTCT risk as trivial (d<0.2), small (0.2≤d<0.5), medium (0.5≤d<0.8), and large (d≥0.8). ALT: alanine aminotransferase; AST: aspartate aminotransferase; HBeAg: hepatitis B e antigen; HBV: hepatitis B virus; MTCT: mother-to-child transmission; PBMC: peripheral blood mononuclear cell.

In the decision tree representation, each node’s split is based on the predictor variables. The result provides several pieces of information that help us interpret the tree. Figure 5 illustrates how the decision trees (ID3/CART) divide the data based on various training-test split ratios (0.95, 0.90, 0.85, 0.80, 0.75, and 0.50), along with the associated MTCT risk classifications (trivial, small, medium, and large) and gain score results. In all splits, HBeAg consistently emerges as the primary splitting factor. The HBeAg-positive branch generally leads to the large risk category when PBMCs are ≥8 × 10^6^ cells/mL, and to medium or small risk when PBMCs are lower. By contrast, HBeAg-negative cases typically split into small risk (when PBMCs are high) or trivial risk (when PBMCs are low). The gain score tables further validate that HBeAg and PBMCs are dominant. Although the precise distributions of risk categories (trivial, small, medium, and large) vary slightly with different splits, the model consistently highlights HBeAg and PBMCs as key factors, reinforcing earlier findings that these 2 variables are essential predictors of mother-to-child HBV transmission risk (Figure 5). According to Figure 6, the risk of MTCT of HBV is stratified based on HBeAg status and PBMC concentration as follows: among HBeAg-positive women, 20 out of 48 training cases with a split ratio of 0.80 (42%) to 27 out of 57 training cases with a split ratio of 0.95 (47%), or 16 out of 30 training cases with a split ratio of 0.50 (53%), were classified as having a high risk of MTCT. Among HBeAg-negative individuals with PBMC concentrations ≥8 × 10^6^ cells/mL, 7 out of 51 training cases with a split ratio of 0.85 (14%) to 8 out of 45 training cases with a split ratio of 0.75 (18%) were categorized as having a small risk. The remaining 21 out of 57 training cases with a split ratio of 0.95 (37%), or 20 out of 53 training cases with a split ratio of 0.90 (38%) to 16 out of 30 training cases with a split ratio of 0.50 (53%) of HBeAg-negative cases with PBMC concentrations <8 × 10^6^ cells/mL were classified as having negligible risk (see Figure 6).

From the dot plot (Figure 4), we can observe several runs of the ID3-based decision tree analysis at different training-test split ratios, indicated in each column. In each run, the algorithm identifies the factor (AST, HBeAg, HBV DNA, or PBMCs) that provides the highest information gain for classification. The y-axis shows the predictive “value” contributed by each factor. The results reveal that HBeAg (green) and PBMCs (purple) frequently demonstrate high information gain, indicating that these 2 factors are dominant in most runs, regardless of the split ratio. By contrast, AST (red) occasionally shows a notable information gain but is less frequent, while HBV DNA (blue) rarely appears as the most critical factor. Overall, these results emphasize that HBeAg and PBMCs are the key factors in the model, remaining consistent across various training-test splits.

In summary, these plots show that HBeAg and PBMCs are typically the most influential factors across different training-test splits, while AST plays a more inconsistent role, and HBV DNA contributes less frequently to classification decisions in these ID3 runs.

### Boosting the Assembly of the Five Factors Shows the Important Groups to Predict the Risk of MTCT

**Figure 7:**
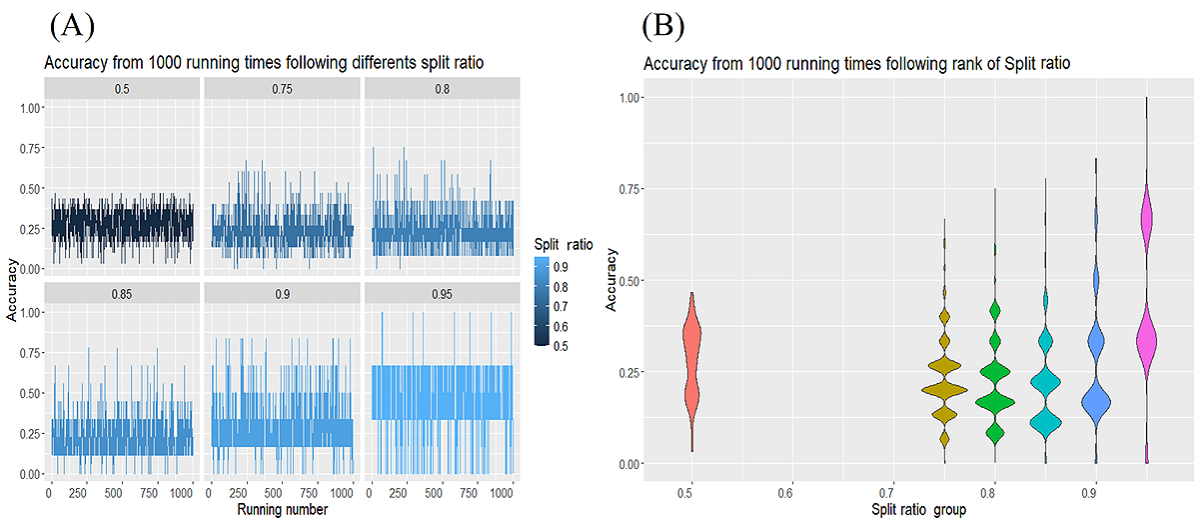
Distribution of accuracy score. (A) Raw distribution. (B) Violin plot shows the median of accuracy.

**Figure 8:**
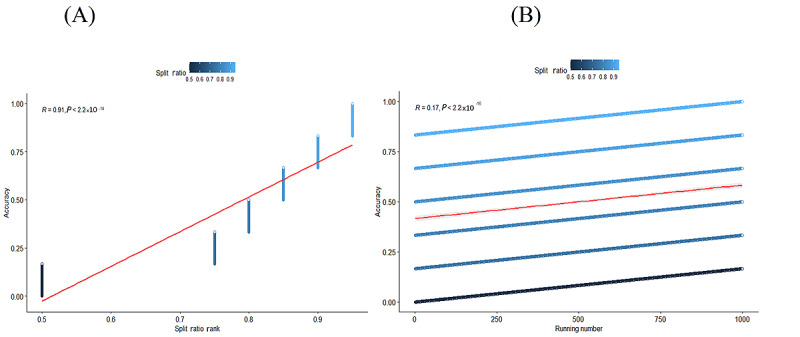
Correlation line between accuracy and split ratio or running number (number of observations). (A) Correlation plot between accuracy and split ratio. (B) Correlation plot between accuracy and running number.

We have 14 cases in which we could enhance the assembly simulation (Multimedia Appendix 11). The split ratio and number of runs influence our predictions for all cases in the group. Figure 6 shows the raw distribution, reflecting the accuracy of each observation in relation to the split ratio. The results of the Pearson chi-square test for the contingency table show that the distribution of risk groups (large, small, and trivial) across different training data split ratios (ranging from 0.75 to 0.95) differs significantly (*χ*^2^_8_=21.16, *P*=.007). When testing each group individually using a 1-way chi-square test, the large group has a *P* value of .002, indicating a clear change in distribution across different training ratios. The small group has a *P* value of .07, which is close to the significance threshold, suggesting a potential trend in distribution change, while the trivial group has a *P* value of .84, indicating a stable distribution that is not significantly affected by the data split ratio. Thus, changes in the train/test ratio may influence how decision trees learn, particularly for groups with distinct characteristics, such as the “large” group. An accuracy measure for classification tasks, using the confusion matrix, provides a better evaluation of classification performance. The general idea is to count how often true instances (true positive and true negative) are misclassified as false (false positive and false negative). We compute the accuracy of the test from the confusion matrix using the following formula: accuracy = (true positive + true negative)/(true positive + true negative + false positive + false negative). The accuracy score reflects the probability that the test data produces the same result as the training data set. Figure 7 shows the results of the correlation test, confirming that the number of observations and the split ratio strongly correlate with accuracy. We repeat this random selection 1000 times for each division ratio, aiming to achieve the highest accuracy. Figure 8 provides an overview of the MTCT risk. Groups 7 and 10 have the highest MTCT risk, with a prevalence ranging from 13 out of 48 (a split ratio of 0.8 from a total of 60 cases, 27%) to 18 out of 54 (a split ratio of 0.9 from a total of 60 cases, 33%). Groups 13 and 14 have the lowest risk, with 9 out of 30 (a split ratio of 0.5 from a total of 60 cases, 30%) to 18 out of 48 (a split ratio of 0.8 from a total of 60 cases, 38%) cases falling into the trivial group, indicating negligible MTCT risk. The other groups show an accuracy ranging from 8 out of 57 (a split ratio of 0.95 from a total of 60 cases, 14%) to 9 out of 30, a split ratio of 0.5 from a total of 60 cases (30%), with cases categorized into the medium- or small-risk groups.

From these plots, we can observe the classification accuracy achieved by the ID3 model over 1000 runs at various training-test split ratios (0.5, 0.75, 0.8, 0.85, 0.9, and 0.95). Each panel corresponds to a specific split ratio, with the y-axis displaying accuracy scores and the x-axis representing the number of runs. Although there is considerable fluctuation within each split ratio, some general patterns emerge. At lower split ratios (eg, 0.5), the model’s accuracy tends to cluster in the lower to mid range (approximately 0.25-0.50). As the split ratio increases (eg, 0.8 or 0.85), accuracy occasionally reaches higher peaks, with some runs exceeding 0.60 or 0.70, though variability remains evident. With even larger training proportions (0.9 and 0.95), the accuracy range broadens further, with some runs achieving relatively high performance while others drop close to 0. Overall, these results indicate that the model’s accuracy is highly sensitive to the specific partitioning of the data set, showing moderate gains and substantial variation as the training-test split ratio changes (Figure 7A).

The violin plots illustrate the distribution of classification accuracy across 1000 runs for each training-test split ratio. On the left, with a split ratio of 0.5, the distribution is relatively narrow, centering around the 0.25-0.40 range. This suggests that using half of the data for training typically results in modest accuracy. As the training proportion increases to 0.75 or 0.8, the distribution shifts upward, revealing higher accuracy values. At split ratios of 0.9 and 0.95, the distribution widens considerably, with some runs achieving very high accuracy—approaching or exceeding 0.75—while others fall closer to 0.20. This suggests that while more training data can improve the model’s performance in some cases, a smaller test set may lead to greater variance, resulting in a wider range of accuracy outcomes (Figure 7B).

Figure 8A shows a strong positive correlation (*R*=0.91, *P*<2.2 × 10^–16^) between the training-test split ratio (on the x-axis) and classification accuracy (on the y-axis). As the amount of data allocated for training increases, the model’s accuracy tends to improve, resulting in a nearly linear upward trend. In Figure 8B, the correlation between the number of runs (indicated on the x-axis) and accuracy is considerably weaker (*R*=0.17, *P*<2.2 × 10^–16^). This suggests that while accuracy shows a slight upward trend over multiple runs, the majority of the variation is better explained by other factors, particularly the split ratio, rather than the order or total number of runs.

**Figure 9:**
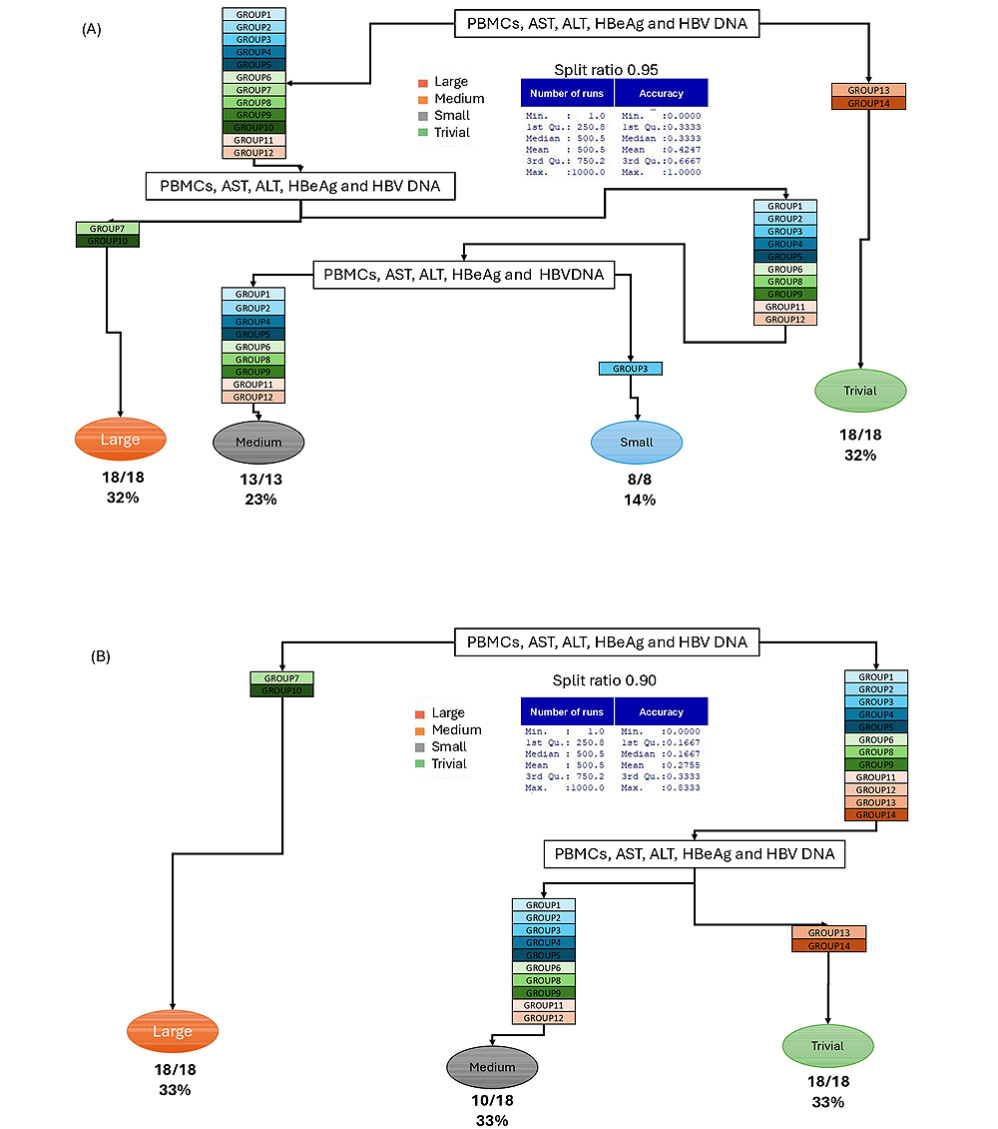
Classification and regression trees with different split ratios. (A) Split ratio 0.95. (B) Split ratio 0.90. Group numbers correspond to those listed in Table 3. The range of mother-to-child transmission (MTCT) risk is based on the Cohen d index. According to Cohen classification, effect sizes on MTCT risk are defined as follows: trivial (d<0.2), small (0.2≤d<0.5), medium (0.5≤d<0.8), and large (d≥0.8). ALT: alanine aminotransferase; AST: aspartate aminotransferase; HBeAg: hepatitis B e antigen; HBV: hepatitis B virus; PBMC: peripheral blood mononuclear cell.

In these CART diagrams (Figure 9 and Multimedia Appendix 13), we observe how the data set is consistently divided by key factors such as PBMCs, AST, ALT, HBeAg, and HBV DNA across various training-test split ratios, ranging from 0.50 to 0.95. Each final branch represents a category of MTCT risk—classified as “large,” “medium,” “small,” or “trivial”—according to the Cohen *d* index. Interestingly, some groups—such as group 7 (with PBMCs < 8 × 10^6^ cells/mL, AST ≥ 14.15 U/L, ALT < 43.34 U/L, HBeAg positive, HBV DNA ≥ 5×10^7^ copies/mL) and group 10 (with PBMCs < 8 × 10^6^ cells/mL, AST ≥ 14.15 U/L, ALT < 43.34 U/L, HBeAg positive, HBV DNA < 5 × 10^7^ copies/mL)—are frequently associated with “very large” effect sizes. By contrast, others—such as group 13 (with PBMCs < 8 × 10^6^ cells/mL, AST ≥ 14.15 U/L, ALT ≥ 43.34 U/L, HBeAg positive, HBV DNA ≥ 5 × 10^7^ copies/mL) and group 14 (with PBMCs < 8 × 10^6^ cells/mL, AST ≥ 14.15 U/L, ALT < 43.34 U/L, HBeAg negative, HBV DNA < 5 × 10^7^ copies/mL)—are categorized as “trivial” in terms of increasing the risk of MTCT. The proportion and number of runs leading to each category are displayed beneath each node. As the training ratio increases, the model becomes more effective at assigning specific groups to their corresponding risk categories, although the overall distribution of runs across large, medium, and trivial categories still fluctuates. This suggests that while certain subgroup characteristics, such as PBMC levels or HBeAg status, influence classification toward high or negligible MTCT risk, the model’s consistency and accuracy are also impacted by how the data are divided for training and testing.

In this study, a “large effect” is defined as a Cohen *d* value of 0.8 or higher. Our decision tree analysis (utilizing ID3 and CART) reveals that the groups identified as 7 and 8 typically show a large effect size, indicating a high risk of MTCT. In these groups, factors such as positive HBeAg status, elevated PBMC concentration (≥8 × 10^6^ cells/mL), and other relevant biochemical markers contribute to an effect size (Cohen *d*) that meets or exceeds the 0.8 threshold. This indicates an increased risk of transmission in these groups.

## Discussion

### Principal Findings

This study aimed to identify key predictors of MTCT of HBV, with a particular focus on maternal HBeAg status and PBMC concentration. We developed and applied an ID3-based decision tree model to analyze clinical data from pregnant Vietnamese women. Our primary finding is that HBeAg positivity, combined with elevated PBMC levels (≥8 × 10^6^ cells/mL), is strongly associated with an increased risk of MTCT. The decision tree model effectively stratified risk based on a combination of virological and biochemical indicators, confirming our hypothesis regarding the predictive value of both PBMCs and HBeAg.

HBV is known to cross the placental barrier, and the presence of maternal HBeAg in newborns at birth supports the occurrence of vertical transmission. Our findings add to the growing body of evidence suggesting that PBMCs may play a critical role in facilitating intrauterine HBV infection [15]. Prior research has shown a 9.5-fold increased risk of HBV infection among neonates who are HBV DNA positive in PBMCs [16]. Similarly, our earlier study involving a cohort of 60 Vietnamese mothers and their infants found a correlation between PBMC concentrations and maternal viral load, particularly in cases with HBV DNA levels below 5 × 10^7^ copies/mL. HBV can be transmitted from mother to child due to its ability to cross the placental barrier. Notably, HBeAg detected in newborns at birth is of maternal origin [17]. In this study, we aim to utilize real-world clinical data to develop a predictive model to determine the risk of MTCT. Our approach uses a machine learning model based on the ID3 decision tree algorithm. The critical decision nodes are determined through a series of calculations, starting from individual (single) entropy to total entropy. Information gain, which quantifies the reduction in entropy, is used to evaluate how effectively a given feature separates or classifies the target outcomes. The feature with the highest information gain is selected as the most informative. This classical entropy-based measure plays a fundamental role in various machine learning algorithms, including decision tree models. In the ID3 algorithm, the attribute selected as the splitting criterion is the one with the highest information gain. This method has proven effective in various machine learning and signal processing applications. Beyond facilitating feature selection, entropy-based measures serve as reliable indicators of data complexity and classification difficulty in real-world scenarios, as demonstrated by Juszczuk et al [18]. Their research underscores the critical importance of using entropy in model construction, especially when dealing with complex biomedical data sets where noise and variability can impact model performance. This supports our application of information gain in identifying PBMC concentration as the most informative variable, aligning both with clinical relevance and theoretical expectations in predicting the risk of MTCT [18]. In machine learning theory, information gain is synonymous with Kullback-Leibler divergence; this index quantifies the amount of information obtained about a random variable by observing another. In the first step, the ID3 algorithm was applied to our data subset without splitting. PBMCs achieved the highest information gain score, which was 0.247 (Multimedia Appendix 13). We could draw a decision tree by choosing PBMCs as the root node, as it has the most significant information gain. From the root node PBMCs, branches extend to other attribute nodes listed in Multimedia Appendix 13. We found that groups 7 and 10 (Multimedia Appendix 11) are associated with a high risk of MTCT, characterized by positive HBeAg status, PBMC serum concentrations above 8 × 10^6^ cells/mL, elevated AST levels (≥14.15 U/L), and low ALT levels (<43.34 U/L). These groups have Cohen *d* values of 1.855 and 1.696, respectively (Multimedia Appendix 11), with associated probabilities ranging from 13 out of 48 (a split ratio of 0.8 from a total of 60 cases, 27%) to 18 out of 54 (a split ratio of 0.9 from a total of 60 cases, 33%), respectively (Figure 9 and Multimedia Appendix 12). The dual roles of HBeAg as both a tolerogen and an immunogen, along with its ability to either suppress or activate the immune response, highlight the complexity of its interactions with the host. Numerous studies have demonstrated that HBeAg can influence both innate and adaptive immune responses, contributing to the persistence of HBV. HBeAg can bind to PBMCs, neutrophils, and B lymphocytes, but not to T lymphocytes. The interaction between HBeAg and monocytes or neutrophils has been shown to be dose dependent, resulting in the inhibition of both cell types. Monocytic myeloid-derived suppressor cells (mMDSCs) are derived from myeloid progenitor cells and account for approximately 0.5% of PBMCs in healthy individuals. The mMDSC population expands during infection, inflammation, and cancer. HBeAg plays a crucial role in the expansion of the mMDSC population and the induction of immune tolerance. Compared with HBeAg-negative patients, HBeAg-positive patients were shown to have significantly higher levels of mMDSCs. When PBMCs from healthy individuals were exposed to HBeAg, there was an increase in mMDSCs and the expression of IL-6 and IL-1β. Additionally, mMDSCs from HBeAg-positive patients suppressed the proliferation of CD4+ and CD8+ T cells. This may represent a potential mechanism by which HBeAg modulates the host immune response during CHB by physically depleting or weakening virus-specific CD4+ and CD8+ T cells. As a result, these cells are unable to proliferate in response to viral antigens or produce essential antiviral and immunostimulatory cytokines, which are crucial for controlling the virus in patients with CHB [14]. Additionally, new research by Padarath et al [14] provided further insights into the various functions of HBeAg and its precursors in the development of chronic HBV infection. According to their review, HBeAg may influence hepatocarcinogenesis through long-term immune modulation and chronic inflammation, in addition to promoting immune evasion and tolerance. This demonstrates that HBeAg serves various functions, including promoting vertical transmission, inhibiting host defenses, and potentially contributing to chronic conditions such as liver cancer. These findings confirm our results, which indicate that HBeAg plays a major role in MTCT risk, and emphasize the need for close monitoring of pregnancies involving HBeAg-positive women. It is well known that both HBeAg and AST are independent risk factors for predicting nonminimal liver inflammation in untreated patients with CHB. In HBeAg-positive untreated patients with CHB, the liver inflammation associated with CHB is linked to the balance between the immune system and HBV infection. Quantitative changes in indicators such as HBsAg and HBeAg can signal the breakdown of immune tolerance and the onset of immune clearance in CHB infection. Including immune-related indicators in the inflammatory prediction model for CHB infection is essential. In general, ALT or AST is included in most models for liver inflammation or fibrosis. It has been found that AST is a better predictor than ALT. HBV DNA, HBsAg, and HBeAg reflect the replication capacity of HBV in untreated patients with CHB. Previous studies have shown that HBsAg and HBeAg are negatively correlated with liver inflammation. In this study, HBsAg, HBeAg, and HBV DNA were all included, with HBeAg demonstrating the best predictive ability. Compared with other models, the significance of this nonminimal liver inflammation model lies in its confirmation of the importance of HBeAg in identifying liver inflammation. On the one hand, HBeAg reflects the replication level of HBV in HBeAg-positive patients with CHB; on the other hand, a decline in HBeAg is often an early sign of the breakdown of immune tolerance [19]. Based on the above calculations, it is evident that further experiments with various random split ratios of the data file are necessary. Different tests will improve prediction performance. The positive correlation between accuracy, division rate, and the number of runs supports this. A clear result is that, after 1000 random runs for each division type, HBeAg and PBMC concentrations consistently show the highest information gain scores. When testing all 5 factors simultaneously, fixed groups emerged in each risk category, highlighting the additive or inhibitory influence of other biochemical indicators on the impact of HBeAg and PBMCs on the risk of MTCT.

Nonetheless, this study has several limitations that should be noted. First, the sample size was relatively small and geographically limited to a single region in Vietnam, which may affect the generalizability of the findings to other populations or ethnicities. Second, while the ID3 decision tree provided valuable insights into variable importance and risk classification, predictive performance could potentially be improved with larger, more heterogeneous data sets, and by comparing it with other, yet untested, machine learning approaches (eg, random forest, XGBoost). Third, additional maternal or fetal factors (eg, nutritional status, coinfections, genetic predisposition) may have been overlooked and could be important, given the constraints of the available data. Finally, the cross-sectional design and retrospective data collection may have introduced bias, limiting the ability to establish a definitive causal relationship. Nevertheless, the findings of the study are still valuable and provide a strong foundation for future research. By identifying key predictors of risk, particularly HBeAg status and PBMC concentration, it was possible to develop a simple yet practical model for predicting risk. These findings represent an essential first step toward developing more comprehensive tools, which can later be refined and validated through larger prospective studies aimed at enhancing clinical decision-making in HBV-infected pregnancies.

Our findings underscore the predictive importance of maternal HBeAg status and PBMC concentration in assessing the risk of HBV MTCT. Incorporating these indicators into clinical screening procedures could enable more targeted interventions during pregnancy. Additionally, our results support the use of machine learning tools in epidemiological risk assessment, particularly in resource-limited settings. Future studies should aim to validate these findings in larger, more diverse cohorts and explore the potential pathways through which PBMCs mediate HBV transmission. These insights may influence decisions regarding the timing and choice of antiviral treatments in high-risk pregnancies, as well as the design of other preventive strategies.

### Limitation

Decision trees are robust and flexible machine learning algorithms. They are interpretable, capable of handling nonlinear relationships, and efficient in computation. Additionally, decision trees can accommodate mixed data types. However, if a small sample is tested, there is a risk of overfitting or overclassification. It is also important to note that only 1 attribute is tested at a time when making a decision.

### Conclusions

This study demonstrated that by combining the ID3 and CART algorithms, data can be interpreted as a decision tree to assist clinicians in their understanding. Additionally, the proposed system provides improved performance by category. The resulting prediction rules, derived from the training data, construct the fastest and most efficient tree. This approach only requires testing enough attributes to classify all the data. By identifying leaf nodes, the test data can be pruned, reducing the number of tests required. The entire data set is explored to construct the tree. This strategy provides clear, structured choices with potential outcomes, making it especially useful in complex diagnostics. It allows health care professionals to review symptoms and test results systematically, supporting more informed decision-making.

## Data Availability

The data sets generated and analyzed during this study are available from the corresponding author on reasonable request.

## Appendix

Multimedia Appendix 1 Relative risk ratio plot. Probability: 1; CBHBsAg positif, 0; and CBHBsAg negatif: 0. CB: cord blood; HBsAg: hepatitis B surface antigen.

Multimedia Appendix 2 Pearson correlation coefficients (R) in the HBV-DNA ≥ 5×10^7^ copies/mL group. The color-coded Pearson correlation coefficients between subclinical indices, including levels of Prothrombin, AST, ALT, RBC, and hemoglobin in maternal blood; the concentration and density of PBMCs; and the status of HBeAg and anti-HBs in both cord and maternal blood. The color of each cell reflects the strength and direction of the correlation, ranging from red (negative correlation) to blue (positive correlation), with intensity corresponding to the magnitude of the association. The correlation strength is indicated by the accompanying color scale. ALT: alanine aminotransferase; AST: aspartate aminotransferase; HBV: hepatitis B virus; HBeAg: hepatitis B e antigen; RBC, red blood cell; PBMC: peripheral blood mononuclear cell.

Multimedia Appendix 3 Pearson correlation coefficients (R values) in the HBV DNA < 5 × 10^7^ copies/mL group are presented in this table. It displays color-coded Pearson correlation coefficients between various subclinical indices, including prothrombin time, AST, ALT, RBC, and hemoglobin levels in maternal blood; the concentration and density of PBMCs; and the status of HBeAg and anti-HBs in both cord and maternal blood. Cell colors indicate the strength and direction of the correlations, ranging from red (negative correlations) to blue (positive correlations), with the intensity corresponding to the magnitude, as shown by the color scale. ALT: alanine aminotransferase; AST: aspartate aminotransferase; HBeAg: hepatitis B e antigen; HBV: hepatitis B virus; RBC: red blood cell; PBMC: peripheral blood mononuclear cell.

Multimedia Appendix 4 *P* values from the Pearson correlation tests in the HBV DNA ≥ 5 × 10^7^ copies/mL group. HBV: hepatitis B virus.

Multimedia Appendix 5 *P* values from the Pearson correlation tests in the HBV DNA < 5 × 10^7^ copies/mL group. HBV: hepatitis B virus.

Multimedia Appendix 6 *P* values from the Fisher exact test comparing the 2 groups: HBV DNA < 5 × 10^7^ copies/mL and HBV DNA ≥ 5 × 10^7^ copies/mL. HBV: hepatitis B virus.

Multimedia Appendix 7 *P* values from the Fisher exact test and Pearson correlation test comparing the correlation coefficients (R) between the 2 groups: HBV DNA < 5 × 10⁷ copies/mL and HBV DNA ≥ 5 × 10^7^ copies/mL. HBV: hepatitis B virus.

Multimedia Appendix 8 Cluster dendrogram of the 2 groups. Left: group with higher maternal viral load (HBV DNA ≥ 5 × 10^7^ copies/mL); right: group with lower maternal viral load (HBV DNA < 5 × 10^7^ copies/mL). The vertical axis represents the Euclidean distance between observations or clusters. Horizontal bars indicate the linkage points at which clusters or individual observations are merged. HBV: hepatitis B virus.

Multimedia Appendix 9 Top: Group with higher viral load (HBV DNA ≥ 5 × 10^7^ copies/mL); bottom: group with lower viral load (HBV DNA < 5 × 10^7^ copies/mL). K-means clustering was optimized using 3 methods: average silhouette, elbow, and gap statistic. The cluster analysis was performed without any missing values. HBV: hepatitis B virus.

Multimedia Appendix 10 Cluster plot. Left: group with higher viral load (HBV DNA ≥ 5 × 10^7^ copies/mL); right: group with lower viral load (HBV DNA < 5 × 10^7^ copies/mL). Clustering was performed with the number of clusters set to 5 and 8, respectively, to visualize the parameter groupings within each viral load group. HBV: hepatitis B virus.

Multimedia Appendix 11 Cohen classified effect sizes on MTCT risk as follows: trivial (d<0.2), small (0.2≤d<0.5), medium (0.5≤d<0.8), and large (d≥0.8). To determine the effect size for each factor, we transformed the natural logarithm of the odds ratio (ln[odds ratio]) by dividing it by 1.81. Additionally, Cohen h was calculated using the formula h = 2 × |arcsin(√p1) − arcsin(√p2)|, where p1 represents the proportion of CBHBsAg-positive cases and p2 the proportion of CBHBsAg-negative cases [9]. The analysis of Cohen h across the groups revealed varying degrees of effect size between CBHBsAg-positive and CBHBsAg-negative proportions. Group 14 demonstrated a very large effect (h=1.46), with a low positive rate (17%) and high negative rate (83%). Group 12 showed a medium effect (h=0.51), indicating a moderate difference. Groups 10 (h=1.70) and 7 (h=1.85) both exhibited very large effects, with positive rates of 88% and 90%, respectively, contrasted with much lower negative rates. Group 13 showed the maximum possible difference (h=3.14), with a 100% positive and 0% negative rate, corresponding to the theoretical upper limit of Cohen h, Π. By contrast, the "other_group" displayed a small effect (h=0.15), with nearly equal proportions of positive and negative cases, suggesting minimal difference between the 2. HBsAg: hepatitis B surface antigen; MTCT: mother-to-child transmission.

Multimedia Appendix 12 Classification and regression trees (CART) with varying split ratios. Panels show CART results with different split ratios: (A) 0.85, (B) 0.80, (C) 0.75, and (D) 0.50. Group numbers correspond to those listed in Table 3. The range of mother-to-child transmission (MTCT) risk is based on Cohen d index. According to the Cohen classification, effect sizes for MTCT risk are defined as follows: trivial (d<0.2), small (0.2≤d<0.5), medium (0.5≤d<0.8), and large (d≥0.8).

Multimedia Appendix 13 The weighted sum of the entropy of the child nodes and the corresponding information gain reaches its maximum value. Cohen classified effect sizes related to mother-to-child transmission risk as follows: trivial (d<0.2), small (0.2≤d<0.5), medium (0.5≤d <0.8), and large (d≥0.8).

## Abbreviations

ALT: alanine aminotransferase
AST: aspartate aminotransferase
CART: classification and regression trees
CHB: chronic hepatitis B
EDTA: ethylenediaminetetraacetic acid
HBeAg: hepatitis B e antigen
HBsAg: hepatitis B surface antigen
HBV: hepatitis B virus
ID3: Iterative Dichotomiser 3
mMDSC: monocytic myeloid-derived suppressor cel
MTCT: mother-to-child transmission
PBMC: peripheral blood mononuclear cell
WHO: World Health Organization
