# Supplementary figures and images for: A Data-Driven Approach to Assessing Hepatitis B Mother-to-Child Transmission Risk Prediction Model: Machine Learning Perspective
Source: JMIR Form Res. 2025 May 23;9:e69838. doi: 10.2196/69838 (PMC12144481; doi:10.2196/69838)

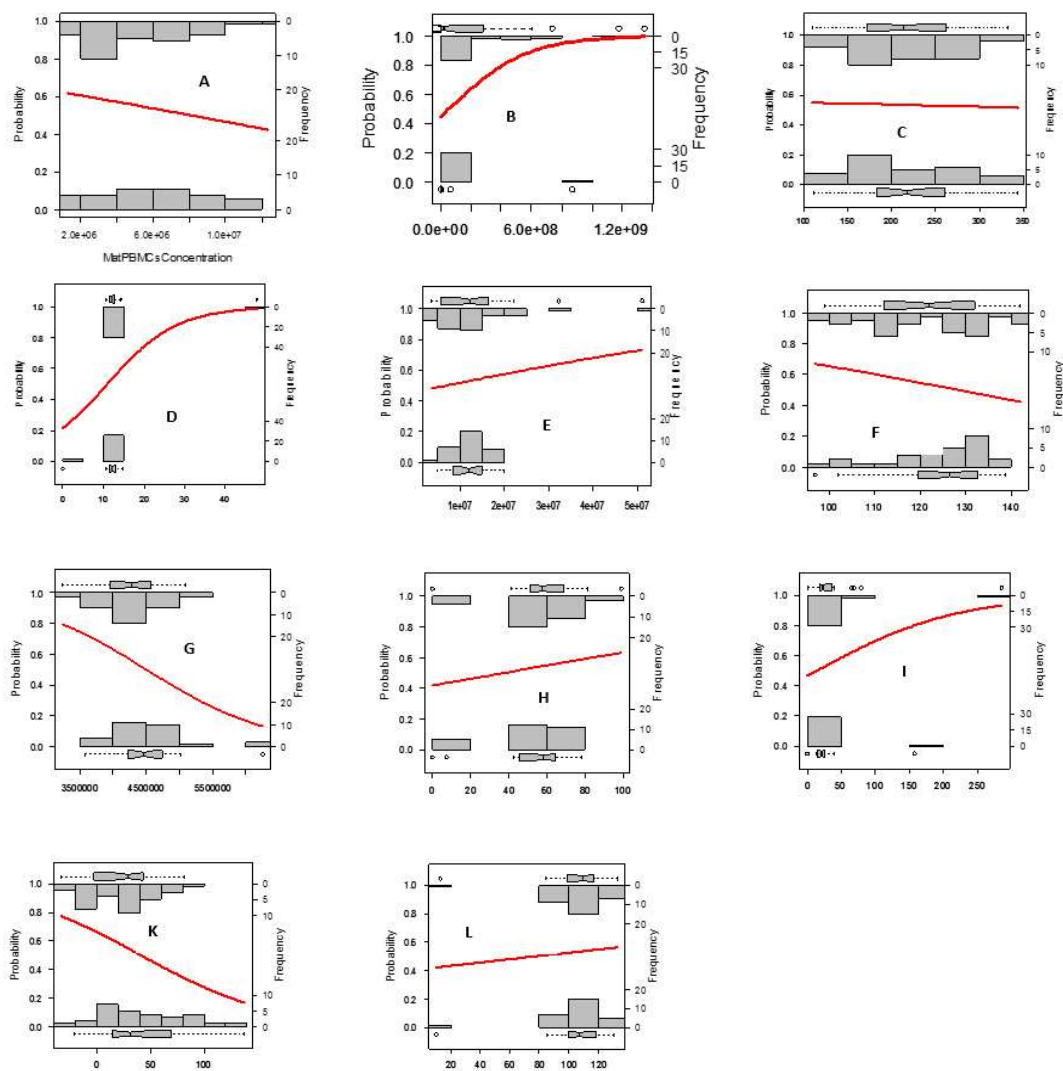

**Supplementary figure 1: Relative Risk Ratio plot.** Probability: 1: CBHBsAg positif, 0: CBHBsAg negatif: 0.

Supplement: Multimedia Appendix 1 [file formative_v9i1e69838_app1.pdf]
