# Supplementary material for: A Data-Driven Approach to Assessing Hepatitis B Mother-to-Child Transmission Risk Prediction Model: Machine Learning Perspective
Source: JMIR Form Res. 2025 May 23;9:e69838. doi: 10.2196/69838 (PMC12144481; doi:10.2196/69838)
Supplement: Multimedia Appendix 3 [file formative_v9i1e69838_app3.pdf]

	MatRBC	MatHb	MatPlatelet	MatProthrombininS	MatProthrombininPPercent	MatAST	MatALT	MatCreatinin	MatBloodProtein	MatAlbuminblood	MatHBeAg	MatAntiHBs	MatHBVDNA	MatPBMCsConcentration	MatPBMCsDensity	CBHBsAg	CBHBeAg	CBAntiHBs	CBAntiHBe	CBMCconcentration	CBMCsDensity
	1	2	3	4	5	6	7	8	9	10	11	12	13	14	15	16	17	18	19	20	21
1	1.00	0.20	-0.11	0.00	-0.07	0.06	0.07	-0.10	0.06	0.08	-0.20	-0.06	0.09	0.02	0.02	-0.30	-0.25	0.07	-0.01	-0.01	0.00
2	0.20	1.00	0.11	0.05	0.05	-0.09	-0.02	-0.04	-0.05	-0.08	-0.05	0.26	0.06	0.07	0.07	-0.14	-0.07	0.25	0.18	-0.03	-0.04
3	-0.11	0.11	1.00	0.14	0.14	-0.10	-0.02	-0.07	0.04	-0.03	0.23	0.06	-0.02	-0.08	-0.08	-0.02	0.11	0.02	0.06	0.12	0.13
4	0.00	0.05	0.14	1.00	-0.75	0.05	0.05	0.15	0.23	0.23	0.22	0.16	0.24	-0.02	-0.02	0.18	0.22	0.07	0.05	-0.11	-0.11
5	-0.07	0.05	0.14	-0.75	1.00	-0.14	-0.08	-0.08	0.00	-0.19	-0.11	0.01	-0.23	0.08	0.08	-0.07	-0.21	-0.17	-0.12	0.05	0.05
6	0.06	-0.09	-0.10	0.05	-0.14	1.00	0.90	0.15	0.12	0.15	0.14	-0.04	0.60	-0.09	-0.09	0.13	0.18	-0.17	-0.05	-0.03	-0.03
7	0.07	-0.02	-0.02	0.05	-0.08	0.90	1.00	0.15	0.19	0.21	0.05	-0.02	0.43	-0.02	-0.02	0.04	0.09	-0.17	-0.04	-0.02	-0.02
8	-0.10	-0.04	-0.07	0.15	-0.08	0.15	0.15	1.00	0.23	0.20	0.01	0.16	0.00	0.27	0.27	0.14	0.01	-0.18	0.03	0.21	0.20
9	0.06	-0.05	0.04	0.23	0.00	0.12	0.19	0.23	1.00	0.75	0.00	0.06	0.06	0.16	0.16	0.11	0.05	-0.09	-0.06	-0.10	-0.12
10	0.08	-0.08	-0.03	0.23	-0.19	0.15	0.21	0.20	0.75	1.00	-0.03	-0.16	0.17	0.20	0.20	0.17	0.09	-0.07	0.06	0.11	0.11
11	-0.20	-0.05	0.23	0.22	-0.11	0.14	0.05	0.01	0.00	-0.03	1.00	0.11	0.50	-0.26	-0.26	0.64	0.80	0.17	0.37	-0.04	-0.02
12	-0.06	0.26	0.06	0.16	0.01	-0.04	-0.02	0.16	0.06	-0.16	0.11	1.00	0.04	-0.14	-0.14	-0.03	0.09	0.25	0.16	-0.05	-0.04
13	0.09	0.06	-0.02	0.24	-0.23	0.60	0.43	0.00	0.06	0.17	0.50	0.04	1.00	-0.29	-0.29	0.39	0.45	0.27	0.44	-0.18	-0.17
14	0.02	0.07	-0.08	-0.02	0.08	-0.09	-0.02	0.27	0.16	0.20	-0.26	-0.14	-0.29	1.00	1.00	-0.07	-0.19	-0.10	-0.05	0.47	0.45
15	0.02	0.07	-0.08	-0.02	0.08	-0.09	-0.02	0.27	0.16	0.20	-0.26	-0.14	-0.29	1.00	1.00	-0.07	-0.19	-0.10	-0.05	0.47	0.45
16	-0.30	-0.14	-0.02	0.18	-0.07	0.13	0.04	0.14	0.11	0.17	0.64	-0.03	0.39	-0.07	-0.07	1.00	0.70	-0.04	0.29	0.00	-0.01
17	-0.25	-0.07	0.11	0.22	-0.21	0.18	0.09	0.01	0.05	0.09	0.80	0.09	0.45	-0.19	-0.19	0.70	1.00	0.29	0.43	0.03	0.05
18	0.07	0.25	0.02	0.07	-0.17	-0.17	-0.17	-0.18	-0.09	-0.07	0.17	0.25	0.27	-0.10	-0.10	-0.04	0.29	1.00	0.77	0.01	0.03
19	-0.01	0.18	0.06	0.05	-0.12	-0.05	-0.04	0.03	-0.06	0.06	0.37	0.16	0.44	-0.05	-0.05	0.29	0.43	0.77	1.00	-0.05	-0.04
20	-0.01	-0.03	0.12	-0.11	0.05	-0.03	-0.02	0.21	-0.10	0.11	-0.04	-0.05	-0.18	0.47	0.47	0.00	0.03	0.01	-0.05	1.00	0.99
21	0.00	-0.04	0.13	-0.11	0.05	-0.03	-0.02	0.20	-0.12	0.11	-0.02	-0.04	-0.17	0.45	0.45	-0.01	0.05	0.03	-0.04	0.99	1.00

Supplementary table 2: R value from Pearson's correlation test in HBVDNA < $5*10^7$ copies/ml group. The color-coded correlation factors between all the subclinical indexes including levels of Prothromibin, AST, ALT, RBC, Hb in mother blood; concentration and density of PBMCs, status of HbeAg, AntiHBs in Cord and Mother blood. The color value of the cells is proportional to the strength of the associations, ranging from red (negative correlations) to blue (positive correlations). The strength of the correlation is indicated in the color scale. Method: Pair-wise Pearson correlation coefficients. Abbreviations: HBV, hepatitis B virus; PBMCs, Peripheral Blood Mononuclear Cells; ALT, Alanine Aminotransferase; AST, Aspartate Aminotransferase; Hb, Hemoglobin; RBC, Red Blood Cell; CBMC, umbilical cord blood mononuclear cells, Mat: Mother or Maternal, CB: Cord blood, HCA: Hierachical cluster analysis, ProthrfombininS: Prothrombin time in second, ProthrombininPercent: Prothrombin % activity.
