# Supplementary material for: A Data-Driven Approach to Assessing Hepatitis B Mother-to-Child Transmission Risk Prediction Model: Machine Learning Perspective
Source: JMIR Form Res. 2025 May 23;9:e69838. doi: 10.2196/69838 (PMC12144481; doi:10.2196/69838)
Supplement: Multimedia Appendix 4 [file formative_v9i1e69838_app4.pdf]

	MatRBC	MatHb	MatPlatelet	MatProthrombininS	MatProthrombininPPercent	MatAST	MatALT	MatCreatinin	MatBloodProtein	MatAlbuminblood	MatAntiHBs	MatHBVDNA	MatPBMCsConcentration	MatPBMCsDensity	CBHBsAg	CBHBeAg	CBAntiHBs	CBAntiHBe	CBMCconcentration	CBMCsDensity
	1	2	3	4	5	6	7	8	9	10	11	12	13	14	15	16	17	18	19	20
1	NA	6.59E-01	9.25E-01	4.27E-01	6.37E-01	5.10E-01	2.29E-01	6.14E-01	5.83E-01	5.39E-01	1.60E-01	3.89E-02	1.24E-02	1.24E-02	6.18E-01	6.18E-01	5.72E-01	5.13E-01	4.02E-01	4.00E-01
2	6.59E-01	NA	3.58E-01	1.55E-02	6.27E-01	5.14E-01	2.18E-01	9.81E-01	1.83E-01	2.15E-01	3.89E-02	6.85E-01	9.50E-01	9.50E-01	4.87E-01	4.87E-01	4.44E-02	4.12E-01	8.97E-03	9.05E-03
3	9.25E-01	3.58E-01	NA	8.26E-01	6.01E-01	7.24E-02	9.14E-02	3.18E-02	1.18E-01	1.40E-01	2.54E-01	3.46E-01	7.60E-01	7.60E-01	5.07E-01	5.07E-01	1.87E-01	6.98E-01	2.25E-01	2.26E-01
4	4.27E-01	1.55E-02	8.26E-01	NA	1.88E-01	4.54E-01	2.27E-01	8.46E-01	6.46E-01	6.85E-01	4.48E-02	4.56E-01	3.93E-01	3.93E-01	1.53E-01	1.53E-01	2.94E-01	6.37E-01	2.41E-02	2.41E-02
5	6.37E-01	6.27E-01	6.01E-01	1.88E-01	NA	6.23E-01	8.39E-01	9.94E-01	5.30E-01	5.31E-01	8.00E-01	9.57E-01	8.22E-01	8.22E-01	3.19E-04	3.19E-04	5.74E-01	6.54E-01	6.27E-01	6.27E-01
6	5.10E-01	5.14E-01	7.24E-02	4.54E-01	6.23E-01	NA	3.03E-03	1.12E-01	7.82E-01	8.33E-01	8.60E-02	2.51E-02	3.60E-01	3.60E-01	7.56E-01	7.56E-01	4.37E-01	6.23E-01	1.17E-01	1.17E-01
7	2.29E-01	2.18E-01	9.14E-02	2.27E-01	8.39E-01	3.03E-03	NA	3.39E-01	6.22E-01	6.85E-01	5.18E-03	1.19E-02	2.21E-01	2.21E-01	8.70E-01	8.70E-01	3.61E-01	5.69E-01	1.75E-02	1.74E-02
8	6.14E-01	9.81E-01	3.18E-02	8.46E-01	9.94E-01	1.12E-01	3.39E-01	NA	2.80E-01	2.80E-01	7.85E-01	5.74E-01	6.92E-01	6.92E-01	7.24E-01	7.24E-01	6.29E-01	7.64E-01	7.11E-01	7.12E-01
9	5.83E-01	1.83E-01	1.18E-01	6.46E-01	5.30E-01	7.82E-01	6.22E-01	2.80E-01	NA	1.57E-07	4.83E-01	6.83E-01	3.29E-01	3.29E-01	5.63E-01	5.63E-01	2.43E-01	5.27E-01	3.00E-01	3.00E-01
10	5.39E-01	2.15E-01	1.40E-01	6.85E-01	5.31E-01	8.33E-01	6.85E-01	2.80E-01	1.57E-07	NA	5.42E-01	6.27E-01	3.09E-01	3.09E-01	5.58E-01	5.58E-01	2.79E-01	5.34E-01	3.44E-01	3.45E-01
11	1.60E-01	3.89E-02	2.54E-01	4.48E-02	8.00E-01	8.60E-02	5.18E-03	7.85E-01	4.83E-01	5.42E-01	NA	6.88E-02	2.10E-01	2.10E-01	7.21E-01	7.21E-01	2.86E-01	7.21E-01	4.19E-04	4.09E-04
12	3.89E-02	6.85E-01	3.46E-01	4.56E-01	9.57E-01	2.51E-02	1.19E-02	5.74E-01	6.83E-01	6.27E-01	6.88E-02	NA	2.98E-02	2.98E-02	9.28E-01	9.28E-01	9.86E-01	3.80E-01	2.05E-01	2.04E-01
13	1.24E-02	9.50E-01	7.60E-01	3.93E-01	8.22E-01	3.60E-01	2.21E-01	6.92E-01	3.29E-01	3.09E-01	2.10E-01	2.98E-02	NA	0.00E+00	8.87E-01	8.87E-01	3.52E-01	1.86E-01	4.55E-01	4.53E-01
14	1.24E-02	9.50E-01	7.60E-01	3.93E-01	8.22E-01	3.60E-01	2.21E-01	6.92E-01	3.29E-01	3.09E-01	2.10E-01	2.98E-02	0.00E+00	NA	8.87E-01	8.87E-01	3.52E-01	1.86E-01	4.55E-01	4.53E-01
15	6.18E-01	4.87E-01	5.07E-01	1.53E-01	3.19E-04	7.56E-01	8.70E-01	7.24E-01	5.63E-01	5.58E-01	7.21E-01	9.28E-01	8.87E-01	8.87E-01	NA	0.00E+00	4.37E-01	7.21E-01	5.29E-01	5.30E-01
16	6.18E-01	4.87E-01	5.07E-01	1.53E-01	3.19E-04	7.56E-01	8.70E-01	7.24E-01	5.63E-01	5.58E-01	7.21E-01	9.28E-01	8.87E-01	8.87E-01	0.00E+00	NA	4.37E-01	7.21E-01	5.29E-01	5.30E-01
17	5.72E-01	4.44E-02	1.87E-01	2.94E-01	5.74E-01	4.37E-01	3.61E-01	6.29E-01	2.43E-01	2.79E-01	2.86E-01	9.86E-01	3.52E-01	3.52E-01	4.37E-01	4.37E-01	NA	2.86E-01	1.23E-01	1.24E-01
18	5.13E-01	4.12E-01	6.98E-01	6.37E-01	6.54E-01	6.23E-01	5.69E-01	7.64E-01	5.27E-01	5.34E-01	7.21E-01	3.80E-01	1.86E-01	1.86E-01	7.21E-01	7.21E-01	2.86E-01	NA	9.47E-01	9.45E-01
19	4.02E-01	8.97E-03	2.25E-01	2.41E-02	6.27E-01	1.17E-01	1.75E-02	7.11E-01	3.00E-01	3.44E-01	4.19E-04	2.05E-01	4.55E-01	4.55E-01	5.29E-01	5.29E-01	1.23E-01	9.47E-01	NA	2.66E-15
20	4.00E-01	9.05E-03	2.26E-01	2.41E-02	6.27E-01	1.17E-01	1.74E-02	7.12E-01	3.00E-01	3.45E-01	4.09E-04	2.04E-01	4.53E-01	4.53E-01	5.30E-01	5.30E-01	1.24E-01	9.45E-01	2.66E-15	NA

Supplementary table 3: p value from Pearson's correlation test in HBVDNA ≥ 5*10^7 copies/ml group. Abbreviations: HBV, hepatitis B virus; PBMCs, Peripheral Blood Mononuclear Cells; ALT, Alanine Aminotransferase; AST, Aspartate Aminotransferase; Hb, Hemoglobin; RBC, Red Blood Cell; CBMC, umbilical cord blood mononuclear cells, Mat: Mother or Maternal, CB: Cord blood, HCA: Hierachical cluster analysis, ProthrfombininS: Prothrombin time in second, ProthrombininPercent: Prothrombin % activity. Signification codes: 0 '***' 0.001 '**' 0.01 '*' 0.05 '.' 0.1 ' ' 1
