# Supplementary material for: A Data-Driven Approach to Assessing Hepatitis B Mother-to-Child Transmission Risk Prediction Model: Machine Learning Perspective
Source: JMIR Form Res. 2025 May 23;9:e69838. doi: 10.2196/69838 (PMC12144481; doi:10.2196/69838)
Supplement: Multimedia Appendix 5 [file formative_v9i1e69838_app5.pdf]

	MatRBC	MatHb	MatPlatelet	MatProthrombininS	MatProthrombininPPercent	MatAST	MatALT	MatCreatinin	MatBloodProtein	MatAlbuminblood	MatHBeAg	MatAntiHBs	MatHBVDNA	MatPBMCsConcentration	MatPBMCsDensity	CBHBsAg	CBHBeAg	CBAntiHBs	CBAntiHBe	CBMCconcentration	CBMCsDensity
	1	2	3	4	5	6	7	8	9	10	11	12	13	14	15	16	17	18	19	20	21
1	NA	1.52E-01	4.50E-01	9.80E-01	6.02E-01	6.51E-01	6.17E-01	4.80E-01	6.64E-01	5.89E-01	1.57E-01	6.57E-01	5.29E-01	8.94E-01	8.91E-01	2.66E-02	7.38E-02	6.05E-01	9.69E-01	9.70E-01	9.87E-01
2	1.52E-01	NA	4.20E-01	7.35E-01	7.42E-01	5.14E-01	8.99E-01	7.74E-01	7.26E-01	5.78E-01	7.48E-01	6.36E-02	6.52E-01	6.41E-01	6.40E-01	3.13E-01	6.25E-01	6.65E-02	2.04E-01	8.46E-01	7.56E-01
3	4.50E-01	4.20E-01	NA	3.20E-01	3.32E-01	4.75E-01	8.66E-01	6.20E-01	8.01E-01	8.42E-01	1.02E-01	6.89E-01	8.74E-01	5.80E-01	5.80E-01	8.86E-01	4.50E-01	9.05E-01	6.61E-01	3.85E-01	3.59E-01
4	9.80E-01	7.35E-01	3.20E-01	NA	1.52E-10	7.19E-01	7.01E-01	2.89E-01	1.02E-01	9.04E-02	1.12E-01	2.58E-01	8.68E-02	9.07E-01	9.07E-01	2.04E-01	1.08E-01	6.15E-01	7.01E-01	4.15E-01	4.13E-01
5	6.02E-01	7.42E-01	3.32E-01	1.52E-10	NA	3.34E-01	5.51E-01	5.68E-01	9.88E-01	1.84E-01	4.21E-01	9.57E-01	9.25E-02	5.61E-01	5.59E-01	6.41E-01	1.28E-01	2.12E-01	3.86E-01	6.99E-01	6.96E-01
6	6.51E-01	5.14E-01	4.75E-01	7.19E-01	3.34E-01	NA	0.00E+00	2.68E-01	3.94E-01	2.76E-01	3.33E-01	7.80E-01	1.61E-06	5.31E-01	5.30E-01	3.56E-01	2.05E-01	2.32E-01	7.42E-01	8.29E-01	8.30E-01
7	6.17E-01	8.99E-01	8.66E-01	7.01E-01	5.51E-01	0.00E+00	NA	2.75E-01	1.66E-01	1.35E-01	7.23E-01	8.92E-01	1.29E-03	9.12E-01	9.12E-01	7.54E-01	5.23E-01	2.16E-01	7.65E-01	8.95E-01	8.95E-01
8	4.80E-01	7.74E-01	6.20E-01	2.89E-01	5.68E-01	2.68E-01	2.75E-01	NA	9.25E-02	1.58E-01	9.44E-01	2.66E-01	9.86E-01	4.87E-02	4.85E-02	3.21E-01	9.67E-01	1.92E-01	8.47E-01	1.34E-01	1.43E-01
9	6.64E-01	7.26E-01	8.01E-01	1.02E-01	9.88E-01	3.94E-01	1.66E-01	9.25E-02	NA	8.41E-11	9.96E-01	6.44E-01	6.67E-01	2.64E-01	2.65E-01	4.13E-01	7.22E-01	5.28E-01	6.93E-01	4.55E-01	4.08E-01
10	5.89E-01	5.78E-01	8.42E-01	9.04E-02	1.84E-01	2.76E-01	1.35E-01	1.58E-01	8.41E-11	NA	8.57E-01	2.57E-01	2.17E-01	1.58E-01	1.59E-01	2.17E-01	5.33E-01	6.36E-01	6.83E-01	4.17E-01	4.52E-01
11	1.57E-01	7.48E-01	1.02E-01	1.12E-01	4.21E-01	3.33E-01	7.23E-01	9.44E-01	9.96E-01	8.57E-01	NA	4.32E-01	1.20E-04	6.15E-02	6.17E-02	2.83E-07	7.22E-13	2.24E-01	6.86E-03	7.95E-01	8.69E-01
12	6.57E-01	6.36E-02	6.89E-01	2.58E-01	9.57E-01	7.80E-01	8.92E-01	2.66E-01	6.44E-01	2.57E-01	4.32E-01	NA	7.74E-01	3.17E-01	3.17E-01	8.18E-01	4.99E-01	6.72E-02	2.45E-01	7.23E-01	7.76E-01
13	5.29E-01	6.52E-01	8.74E-01	8.68E-02	9.25E-02	1.61E-06	1.29E-03	9.86E-01	6.67E-01	2.17E-01	1.20E-04	7.74E-01	NA	3.49E-02	3.48E-02	3.52E-03	6.82E-04	5.05E-02	9.41E-04	1.99E-01	2.17E-01
14	8.94E-01	6.41E-01	5.80E-01	9.07E-01	5.61E-01	5.31E-01	9.12E-01	4.87E-02	2.64E-01	1.58E-01	6.15E-02	3.17E-01	3.49E-02	NA	0.00E+00	6.27E-01	1.74E-01	4.94E-01	7.32E-01	3.43E-04	7.36E-04
15	8.91E-01	6.40E-01	5.80E-01	9.07E-01	5.59E-01	5.30E-01	9.12E-01	4.85E-02	2.65E-01	1.59E-01	6.17E-02	3.17E-01	3.48E-02	0.00E+00	NA	6.26E-01	1.73E-01	4.93E-01	7.32E-01	3.41E-04	7.33E-04
16	2.66E-02	3.13E-01	8.86E-01	2.04E-01	6.41E-01	3.56E-01	7.54E-01	3.21E-01	4.13E-01	2.17E-01	2.83E-07	8.18E-01	3.52E-03	6.27E-01	6.26E-01	NA	5.34E-09	7.73E-01	3.37E-02	9.76E-01	9.31E-01
17	7.38E-02	6.25E-01	4.50E-01	1.08E-01	1.28E-01	2.05E-01	5.23E-01	9.67E-01	7.22E-01	5.33E-01	7.22E-13	4.99E-01	6.82E-04	1.74E-01	1.73E-01	5.34E-09	NA	3.75E-02	1.48E-03	8.05E-01	7.40E-01
18	6.05E-01	6.65E-02	9.05E-01	6.15E-01	2.12E-01	2.32E-01	2.16E-01	1.92E-01	5.28E-01	6.36E-01	2.24E-01	6.72E-02	5.05E-02	4.94E-01	4.93E-01	7.73E-01	3.75E-02	NA	1.94E-11	9.20E-01	8.36E-01
19	9.69E-01	2.04E-01	6.61E-01	7.01E-01	3.86E-01	7.42E-01	7.65E-01	8.47E-01	6.93E-01	6.83E-01	6.86E-03	2.45E-01	9.41E-04	7.32E-01	7.32E-01	3.37E-02	1.48E-03	1.94E-11	NA	7.43E-01	7.71E-01
20	9.70E-01	8.46E-01	3.85E-01	4.15E-01	6.99E-01	8.29E-01	8.95E-01	1.34E-01	4.55E-01	4.17E-01	7.95E-01	7.23E-01	1.99E-01	3.43E-04	3.41E-04	9.76E-01	8.05E-01	9.20E-01	7.43E-01	NA	0.00E+00
21	9.87E-01	7.56E-01	3.59E-01	4.13E-01	6.96E-01	8.30E-01	8.95E-01	1.43E-01	4.08E-01	4.52E-01	8.69E-01	7.76E-01	2.17E-01	7.36E-04	7.33E-04	9.31E-01	7.40E-01	8.36E-01	7.71E-01	0.00E+00	NA

Supplementary table 4: p value from Pearson's correlation test in HBVDNA < $5*10^7$ copies/ml group. Abbreviations: HBV, hepatitis B virus; PBMCs, Peripheral Blood Mononuclear Cells; ALT, Alanine Aminotransferase; AST, Aspartate Aminotransferase; Hb, Hemoglobin; RBC, Red Blood Cell; CBMC, umbilical cord blood mononuclear cells, Mat: Mother or Maternal, CB: Cord blood, HCA: Hierachical cluster analysis, ProthrfombininS: Prothrombin time in second, ProthrombininPercent: Prothrombin % activity. Signification codes: 0 '***' 0.001 '**' 0.01 '*' 0.05 '.' 0.1 ' ' 1
