# Supplementary material for: A Data-Driven Approach to Assessing Hepatitis B Mother-to-Child Transmission Risk Prediction Model: Machine Learning Perspective
Source: JMIR Form Res. 2025 May 23;9:e69838. doi: 10.2196/69838 (PMC12144481; doi:10.2196/69838)
Supplement: Multimedia Appendix 6 [file formative_v9i1e69838_app6.pdf]

	F value	Pr(>F)	Signification code
MatRBC	1.3	0.26	
MatHb	0.29	0.59	
MatPlatelet	0.41	0.52	
MatProthrombininS	0.11	0.73	
MatProthrombininPPercent	1.42	0.24	
MatAST	0.24	0.63	
MatALT	1.65	0.20	
MatCreatinin	0.07	0.80	
MatBloodProtein	0.37	0.55	
MatAlbumiinblood	0.14	0.70	
MatHBeAg	na		
MatAntiHBs	1.33	0.25	
MatPBMCsConcentration	0.02	0.89	
MatPBMCsDensity	0.02	0.89	
CBHBsAg	3.41	0.07	.
CBAntiHBs	0.70	0.41	
CBAntiHBe	8.32	0.006	**
CBMCconcentration	1.10	0.30	
CBMCsDensity	1.17	0.28	

Supplementary table 5: p value Fisher test between two groups, HBVDNA < 5*10^7 copies/ml and HBVDNA ≥ 5*10^7 copies/ml. Abbreviations: HBV, hepatitis B virus; PBMCs, Peripheral Blood Mononuclear Cells; ALT, Alanine Aminotransferase; AST, Aspartate Aminotransferase; Hb, Hemoglobin; RBC, Red Blood Cell; CBMC, umbilical cord blood mononuclear cells, Mat: Mother or Maternal, CB: Cord blood, HCA: Hierachical cluster analysis, ProthrfombininS: Prothrombin time in second, ProthrombininPercent: Prothrombin % activity. Signification codes: 0 ‘***’ 0.001 ‘**’ 0.01 ‘*’ 0.05 ‘.’ 0.1 ‘ ’ 1
