# Supplementary material for: A Data-Driven Approach to Assessing Hepatitis B Mother-to-Child Transmission Risk Prediction Model: Machine Learning Perspective
Source: JMIR Form Res. 2025 May 23;9:e69838. doi: 10.2196/69838 (PMC12144481; doi:10.2196/69838)
Supplement: Multimedia Appendix 7 [file formative_v9i1e69838_app7.pdf]

	**F value**	**Pr(>F)**	Signification code	t (Pearson's Test)	df (Pearson's Test)	p-value(Pearson's Test)	Signification code	cor	95%CI	
									lower	upper
MatRBC	0.02	0.89		2.17	18	0.04	.	0.46	0.017	0.75
MatHb	12.37	0.001	**.	2.58	18	0.02	*	0.52	0.1	0.78
MatPlatelet	2.83	0.1		1.45	18	0.17		0.32	-0.14	0.67
MatProthrombininS	2.68	0.10		0.69	18	0.50		0.16	-0.30	0.56
MatProthrombininPPercent	4.12	0.05	*	0.58	18	0.56		0.14	-0.33	0.55
MatAST	1.12	0.30		2.3	18	0.03	*	0.47	0.04	0.76
MatALT	0.97	0.33		1.86	18	0.07	.	0.40	-0.05	0.72
MatCreatinin	0.41	0.53		2.15	18	0.05	.	0.45	0.01	0.74
MatBloodProtein	0.61	0.44		2.71	18	0.01	*	0.54	0.13	0.79
MatAlbumiinblood	0.23	0.64		2.93	18	0.009	**	0.57	0.17	0.81
MatAntiHBs	2.54	0.12		1.07	18	0.30		0.24	-0.22	0.62
MatPBMCsConcentration	0.65	0.42		3.46	18	0.003	**	0.63	0.26	0.84
MatPBMCsDensity	0.65	0.42		3.46	18	0.003	**	0.63	0.26	0.84
CBHBsAg	0.22	0.64		2.72	18	0.014	*	0.54	0.13	0.79
CBAntiHBs	5.08	0.03	*	1.32	18	0.20		0.30	-0.17	0.65
CBAntiHBe	0.12	0.73		2.47	18	0.024	*	0.5	0.077	0.77
CBMCconcentration	2.24	0.14		0.73	18	0.47		0.17	-0.29	0.57
CBMCsDensity	2.29	0.14		0.81	18	0.43		0.19	-0.28	0.58

Supplementary table 6: **p value Fisher test and Pearson's Test between the R from two groups, HBVDNA < 5*10^7 copies/ml and HBVDNA ≥ 5*10^7 copies/ml.** Abbreviations: HBV, hepatitis B virus; PBMCs, Peripheral Blood Mononuclear Cells; ALT, Alanine Aminotransferase; AST, Aspartate Aminotransferase; Hb, Hemoglobin; RBC, Red Blood Cell; CBMC, umbilical cord blood mononuclear cells, Mat: Mother or Maternal, CB: Cord blood, HCA: Hierachical cluster analysis, ProthrfombininS: Prothrombin time in second, ProthrombininPercent: Prothrombin % activity. Signification codes: 0 '***' 0.001 '**' 0.01 '*' 0.05 '.' 0.1 ' ' 1
