# Supplementary material for: A Data-Driven Approach to Assessing Hepatitis B Mother-to-Child Transmission Risk Prediction Model: Machine Learning Perspective
Source: JMIR Form Res. 2025 May 23;9:e69838. doi: 10.2196/69838 (PMC12144481; doi:10.2196/69838)
Supplement: Multimedia Appendix 8 [file formative_v9i1e69838_app8.pdf]

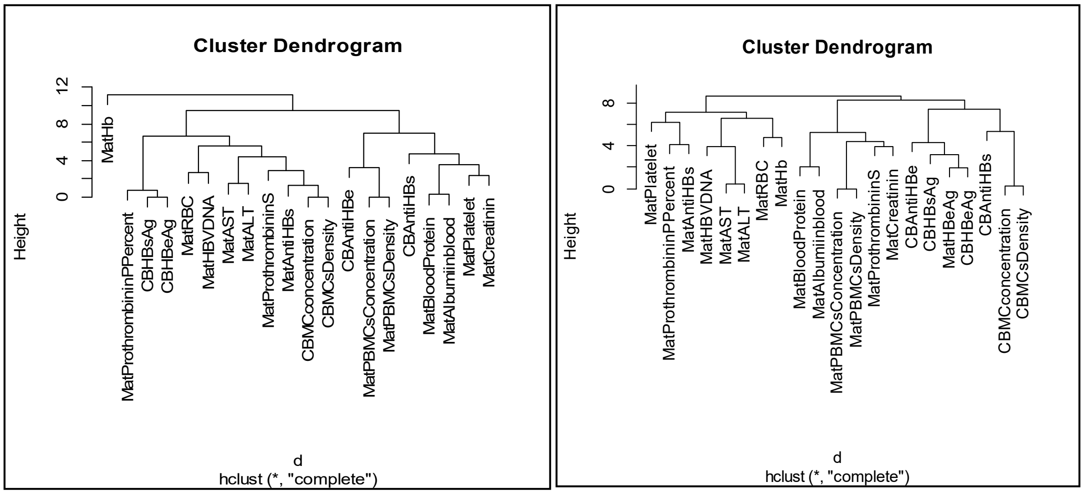

Supplemetary Figure 2: Cluster Drendrogram of two groups. (left) group of higher maternal viral load ([HBV DNA] ≥ 5*10^7copies/ml), (right) group of lower maternal viral load ([HBV DNA] < 5*10^7copies/ml). The height axis displays the Euclidean distance between observations and/or clusters. The horizontal bars indicate the point at which two clusters/observations are merged. HBV, hepatitis B virus; PBMCs, Peripheral Blood Mononuclear Cells; ALT, Alanine Aminotransferase; AST, Aspartate Aminotransferase; Hb, Hemoglobin; RBC, Red Blood Cell; CBMC, umbilical cord blood mononuclear cells, Mat: Mother or Maternal, CB: Cord blood, HCA: Hierachical cluster analysis, ProthrfombininS: Prothrombin time in second, ProthrombininPercent: Prothrombin % activity.
