# Supplementary material for: A Data-Driven Approach to Assessing Hepatitis B Mother-to-Child Transmission Risk Prediction Model: Machine Learning Perspective
Source: JMIR Form Res. 2025 May 23;9:e69838. doi: 10.2196/69838 (PMC12144481; doi:10.2196/69838)
Supplement: Multimedia Appendix 9 [file formative_v9i1e69838_app9.pdf]

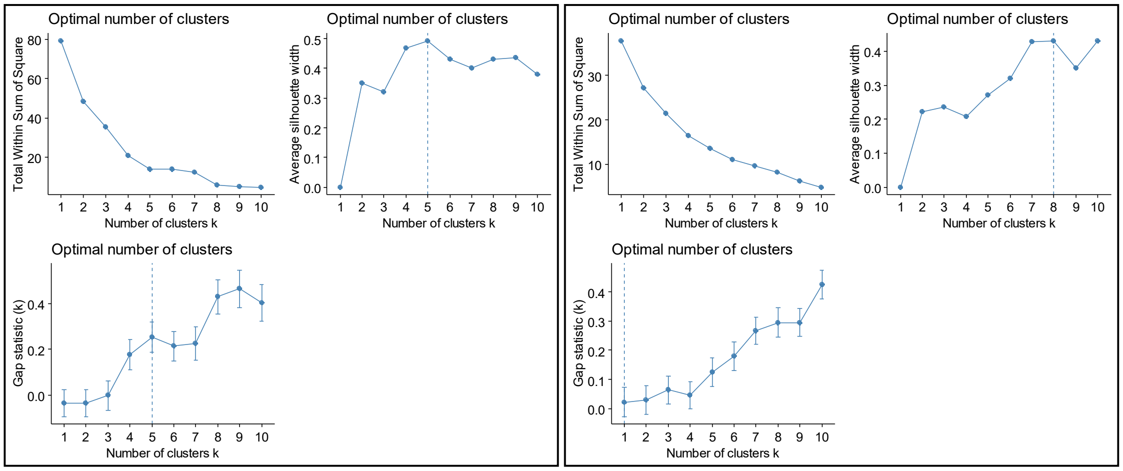

Supplemetary Figure 3: Optimal number of clusters. (upper) group of higher viral load ([HBV DNA] ≥ 5*10^7copies/ml), (lower) group of lower viral load ([HBV DNA] < 5*10^7copies/ml). K-means was optimized following three methods: Avarage Silhouette, Elbow and Gap statistic. The cluster analysis carried out did not have any missing values.
