# Supplementary material for: A Data-Driven Approach to Assessing Hepatitis B Mother-to-Child Transmission Risk Prediction Model: Machine Learning Perspective
Source: JMIR Form Res. 2025 May 23;9:e69838. doi: 10.2196/69838 (PMC12144481; doi:10.2196/69838)
Supplement: Multimedia Appendix 11 [file formative_v9i1e69838_app11.docx]

| Group | PBMCs  (cells/ml) | AST (U/L) | ALT (U/L) | HBeAg | HBVDNA (copies/ml) | freq Total | freg CBHBsAg_pos | freg CBHBsAg_neg | Effect increases the MTCT | Effect decreases the MTCT | prop_pos | prop_neg | cohen_h | OR | 95%-CI | RR | 95%-CI | pval |
| --- | --- | --- | --- | --- | --- | --- | --- | --- | --- | --- | --- | --- | --- | --- | --- | --- | --- | --- |
| 14 | < 8x10^6^ | ≥ 14.15 | < 43.34 | negative | < 5x10^7^ | 18 | 3 | 15 | Trivial | Very large | 0.167 | 0.833 | 1.459 | 0.04 | [0.0069; 0.2309] | 0.20 | [0.0697; 0.5735] | 0.0002 |
| 12 | ≥ 8x10^6^ | ≥ 14.15 | < 43.34 | negative | < 5x10^7^ | 8 | 3 | 5 | Small/medium | Medium | 0.375 | 0.625 | 0.505 | 0.36 | [0.0476; 2.7253] | 0.60 | [0.2114; 1.7031] | 0.6190 |
| 10 | < 8x10^6^ | ≥ 14.15 | < 43.34 | positive | < 5x10^7^ | 8 | 7 | 1 | Very large/large | trivial | 0.875 | 0.125 | 1.696 | 49.00 | [2.5310; 948.6191] | 7.00 | [1.0985; 44.6075] | 0.0101 |
| 7 | < 8x10^6^ | ≥ 14.15 | < 43.34 | positive | ≥ 5x10^7^ | 10 | 9 | 1 | Very large/large | trivial | 0.900 | 0.100 | 1.855 | 81.00 | [4.3610; 1504.4625] | 9.00 | [1.3860; 58.4430] | 0.0011 |
| 13 | < 8x10^6^ | ≥ 14.15 | ≥ 43.34 | positive | ≥ 5x10^7^ | 3 | 3 | 0 | trivial | Maximum | 1.000 | 0.000 | 3.142 | 49.00 | [0.7417; 3236.9913] | 7.00 | [0.5378; 91.1134] | 0.1000 |
|  | All other group | | | | | 13 | 7 | 6 | medium | Small | 0.538 | 0.462 | 0.154 | 1.36 | [0.2912; 6.3623] | 1.17 | [0.5384; 2.5281] | 1.0000 |
| 1 | < 8x10^6^ | < 14.15 | < 43.34 | negative | < 5x10^7^ | 4 | 1 | 3 |  |  |  |  |  |  |  |  |  |  |
| 2 | < 8x10^6^ | < 14.15 | < 43.34 | positive | < 5x10^7^ | 1 | 1 | 0 |  |  |  |  |  |  |  |  |  |  |
| 3 | < 8x10^6^ | < 14.15 | < 43.34 | positive | ≥ 5x10^7^ | 1 | 1 | 0 |  |  |  |  |  |  |  |  |  |  |
| 6 | < 8x10^6^ | ≥ 14.15 | ≥ 43.34 | negative | < 5x10^7^ | 1 | 1 | 0 |  |  |  |  |  |  |  |  |  |  |
| 8 | < 8x10^6^ | ≥ 14.15 | ≥ 43.34 | negative | ≥ 5x10^7^ | 1 | 0 | 1 |  |  |  |  |  |  |  |  |  |  |
| 5 | ≥ 8x10^6^ | < 14.15 | < 43.34 | negative | < 5x10^7^ | 1 | 0 | 1 |  |  |  |  |  |  |  |  |  |  |
| 11 | ≥ 8x10^6^ | < 14.15 | < 43.34 | positive | < 5x10^7^ | 1 | 0 | 1 |  |  |  |  |  |  |  |  |  |  |
| 9 | ≥ 8x10^6^ | ≥ 14.15 | < 43.34 | positive | < 5x10^7^ | 1 | 1 | 0 |  |  |  |  |  |  |  |  |  |  |
| 4 | ≥ 8x10^6^ | ≥ 14.15 | < 43.34 | positive | ≥ 5x10^7^ | 2 | 2 | 0 |  |  |  |  |  |  |  |  |  |  |

**Multimedia Appendix 12: Risk ratio and Cohen's index result.** Cohen classified effect sizes on MTCT risk as trivial (d<0.2), small (0.2 ≤ d < 0.5), medium (0.5 ≤ d < 0.8), and large (d ≥ 0.8). To determine the effect size for each factor, we transformed the natural logarithm of the odds ratio (ln(odds ratio)) by dividing it by 1.81, based on their odds ratios. Formular Cohen’s h is: $h=2\times\mid arcsin(\sqrt{p1}\boldsymbol{)}-arcsin(\sqrt{p2}\boldsymbol{)\mid}$ , in there: p1: is proportion of CBHBsAg positive, p2: is proportion of CBHBsAg negative [9]. The analysis of Cohen's *h* across the groups reveals varying degrees of effect size between the proportions of CBHBsAg-positive and negative cases. Group 14 demonstrates a **very large effect** (*h* = 1.46), characterized by a notably low proportion of positive cases (17%) and a high proportion of negative cases (83%). Group 12 shows a **medium effect** (*h* = 0.51), indicating a moderate difference between the two proportions. Groups 10 (*h* = 1.70) and 7 (*h* = 1.85) both reflect **very large effects**, with high positive rates of 88% and 90% respectively, contrasted with much lower negative rates. Group 13 exhibits the **maximum possible difference** (*h* = 3.14), where the proportion of positives is 100% and negatives 0%, corresponding to the theoretical limit of Cohen’s *h*, π. In contrast, the *other_group* shows a **small effect** (*h* = 0.15), with nearly equal positive and negative proportions, suggesting minimal difference between the two groups.
