# Supplementary material for: A Data-Driven Approach to Assessing Hepatitis B Mother-to-Child Transmission Risk Prediction Model: Machine Learning Perspective
Source: JMIR Form Res. 2025 May 23;9:e69838. doi: 10.2196/69838 (PMC12144481; doi:10.2196/69838)
Supplement: Multimedia Appendix 12 [file formative_v9i1e69838_app12.pdf]

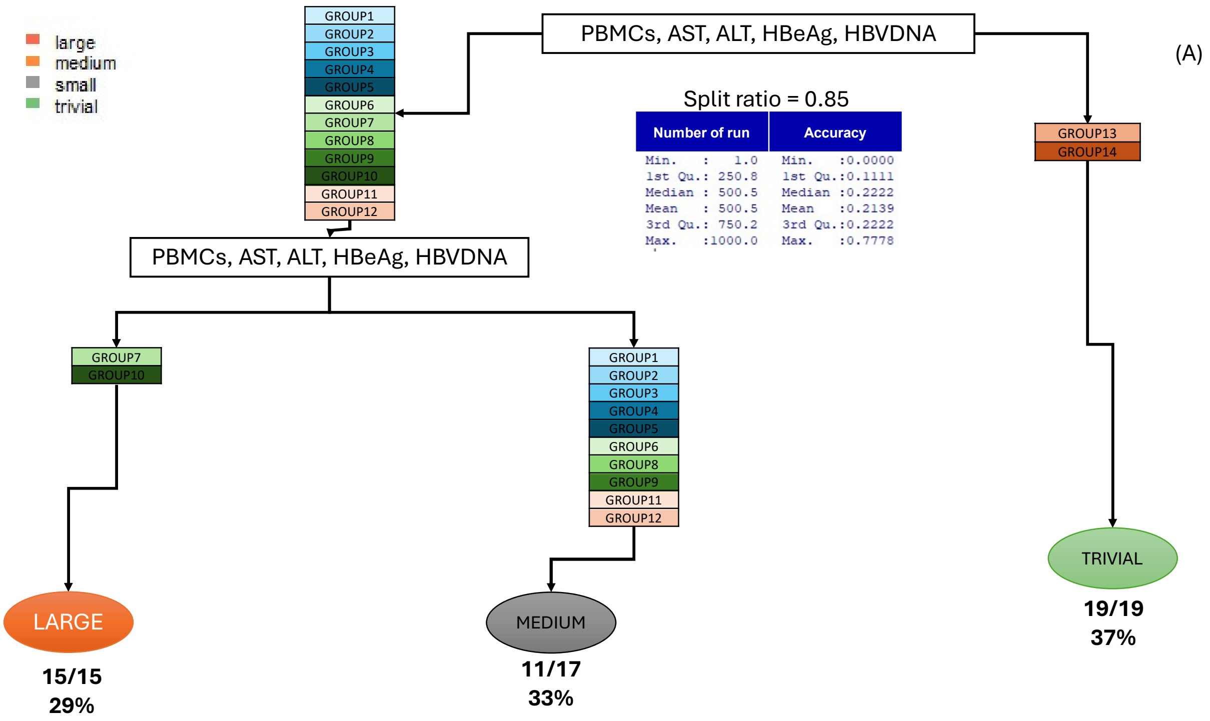
large
medium
small
trivial
PBMCs, AST, ALT, HBeAg, HBVDNA
(A)
GROUP1
GROUP2
GROUP3
GROUP4
GROUP5
GROUP6
GROUP7
GROUP8
GROUP9
GROUP10
GROUP11
GROUP12
Split ratio = 0.85
Number of run
Accuracy
Min. : 1.0
1st Qu.: 250.8
Median : 500.5
Mean : 500.5
3rd Qu.: 750.2
Max. :1000.0
Min. :0.0000
1st Qu.:0.1111
Median :0.2222
Mean :0.2139
3rd Qu.:0.2222
Max. :0.7778
GROUP13
GROUP14
PBMCs, AST, ALT, HBeAg, HBVDNA
GROUP7
GROUP10
GROUP1
GROUP2
GROUP3
GROUP4
GROUP5
GROUP6
GROUP8
GROUP9
GROUP11
GROUP12
TRIVIAL
19/19
37%
LARGE
15/15
29%
MEDIUM
11/17
33%

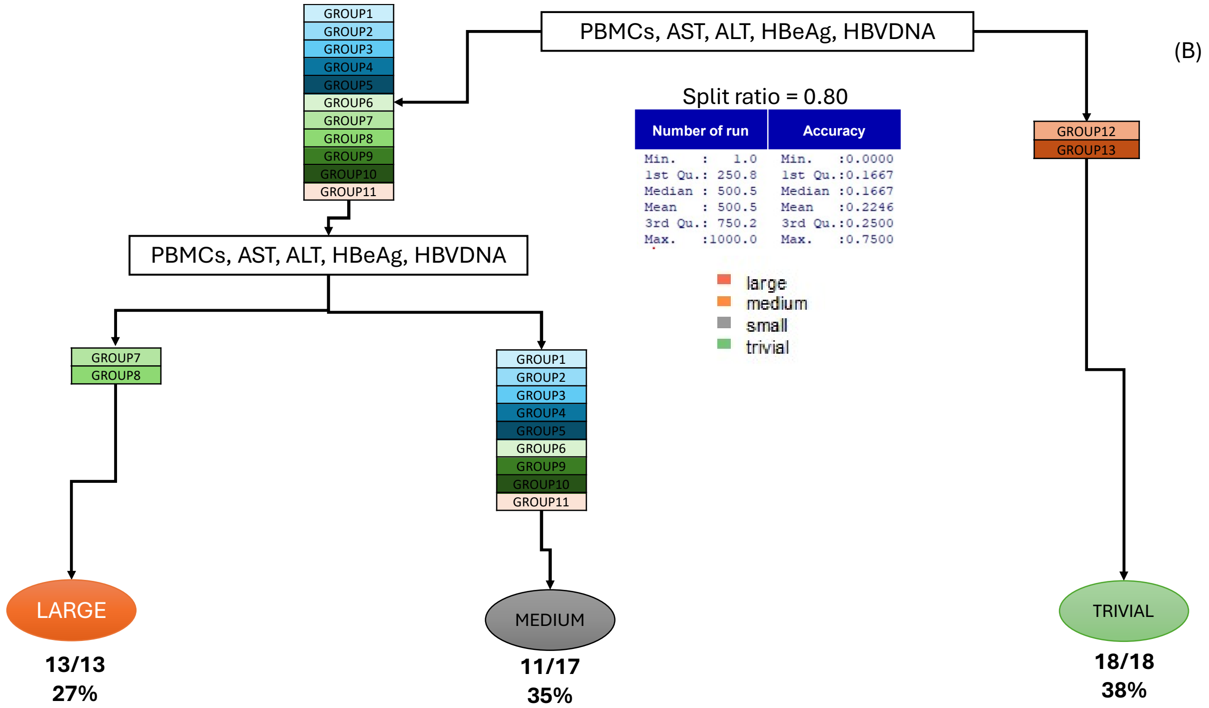
PBMCs, AST, ALT, HBeAg, HBVDNA
(B)
GROUP1
GROUP2
GROUP3
GROUP4
GROUP5
GROUP6
GROUP7
GROUP8
GROUP9
GROUP10
GROUP11
Split ratio = 0.80
Number of run
Accuracy
Min. : 1.0
1st Qu.: 250.8
Median : 500.5
Mean : 500.5
3rd Qu.: 750.2
Max. :1000.0
Min. :0.0000
1st Qu.:0.1667
Median :0.1667
Mean :0.2246
3rd Qu.:0.2500
Max. :0.7500
GROUP12
GROUP13
PBMCs, AST, ALT, HBeAg, HBVDNA
large
medium
small
trivial
GROUP7
GROUP8
GROUP1
GROUP2
GROUP3
GROUP4
GROUP5
GROUP6
GROUP9
GROUP10
GROUP11
LARGE
13/13
27%
MEDIUM
11/17
35%
TRIVIAL
18/18
38%

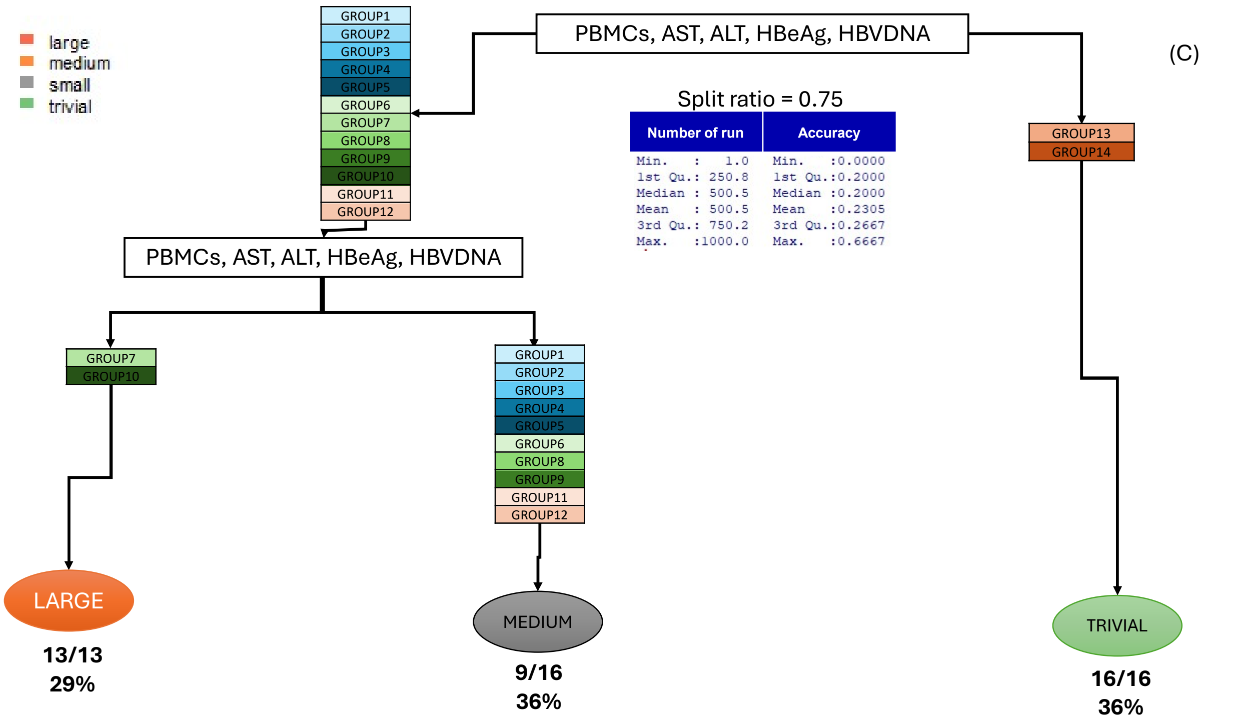

Split ratio = 0.75

Number of run	Accuracy
Min. : 1.0	Min. :0.0000
1st Qu.: 250.8	1st Qu.:0.2000
Median : 500.5	Median :0.2000
Mean : 500.5	Mean :0.2305
3rd Qu.: 750.2	3rd Qu.:0.2667
Max. :1000.0	Max. :0.6667

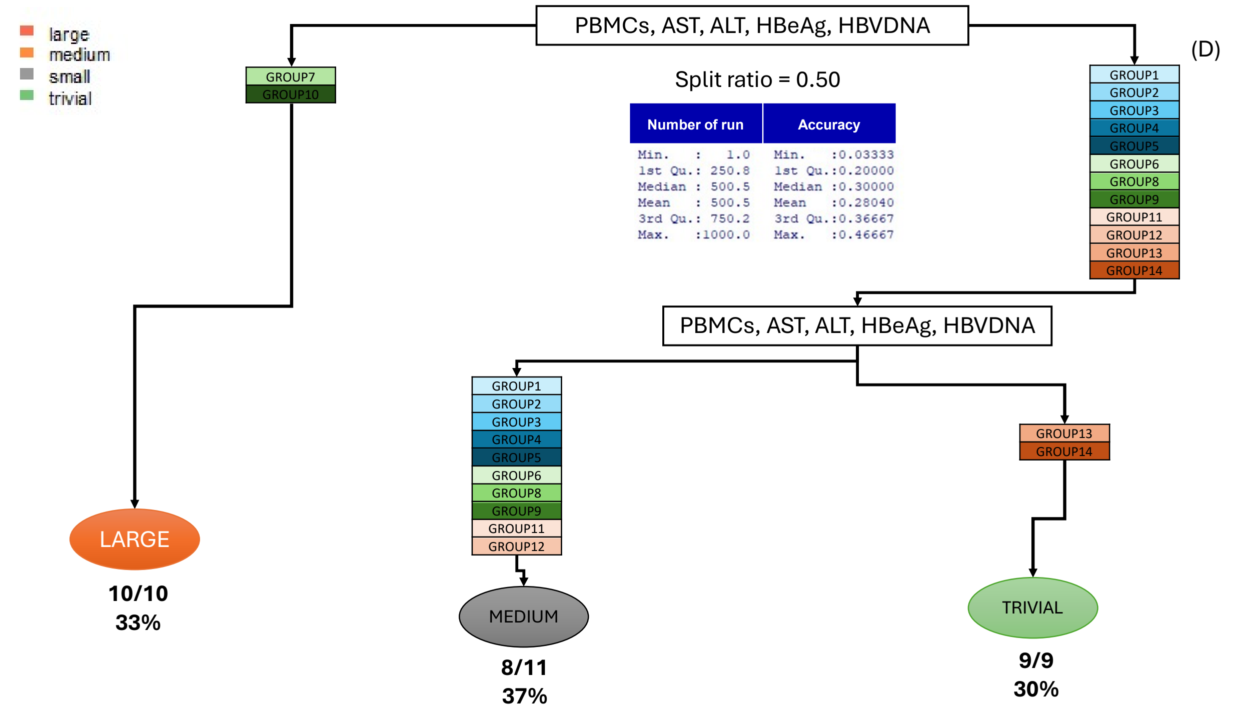

Split ratio = 0.50

Number of run	Accuracy
Min. : 1.0	Min. :0.03333
1st Qu.: 250.8	1st Qu.:0.20000
Median : 500.5	Median :0.30000
Mean : 500.5	Mean :0.28040
3rd Qu.: 750.2	3rd Qu.:0.36667
Max. :1000.0	Max. :0.46667

Supplemetary Figure 5: Classification And Regression Tree (CART) with the split ratio (A): split ratio = 0.85, (B): split ratio = 0.80, (C): split ratio = 0.75, (D): split ratio = 0.50. The group is numbered following the table 3. Range of MTCT risk is from Cohen's index. Cohen classified effect sizes on MTCT risk as trivial ($d<0.2$), small ($0.2 \leq d < 0.5$), medium ($0.5 \leq d < 0.8$), and large ($d \geq 0.8$).
