# Supplementary material for: A Data-Driven Approach to Assessing Hepatitis B Mother-to-Child Transmission Risk Prediction Model: Machine Learning Perspective
Source: JMIR Form Res. 2025 May 23;9:e69838. doi: 10.2196/69838 (PMC12144481; doi:10.2196/69838)
Supplement: Multimedia Appendix 13 [file formative_v9i1e69838_app13.docx]

| Attributes | Values | *Childnode* | The number of points in each child node | | N trivial | N small | N medium | N large | Entropy (equation (2) and (3) | | The total entropy weight (Equation (4)) | | information gain (Equation (5)) |
| --- | --- | --- | --- | --- | --- | --- | --- | --- | --- | --- | --- | --- | --- |
| HBeAg | Positive | ***S****_p_* | *m_p_* | 7 | 1 | 1 | 5 | 0 | 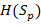 | 1.157 | 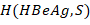 | 1.154 | 0.166 |
|  | Negative | ***S****_n_* | *m_n_* | 10 | 1 | 0 | 7 | 2 | 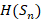 | 1.149 |  |  |  |
| ALT(U/L) | <43.34 | ***S****_altlow_* | *m_altlow_* | 12 | 1 | 1 | 8 | 12 | 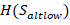 | 1.42 | 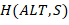 | 1.243 | 0.05 |
|  | ≥43.34 | ***S****_althigh_* | *m_althigh_* | 5 | 1 | 0 | 4 | 0 | 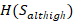 | 0.72 |  |  |  |
| AST (U/L) | *<14.15* | ***S****_astlow_* | *m_astlow_* | 5 | 5 | 0 | 0 | 0 | 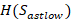 | 0 | 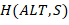 | 1.139 | 0.184 |
|  | *≥14.15* | ***S****_asthigh_* | *m_asthigh_* | 12 | 2 | 1 | 7 | 2 | 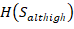 | 1.61 |  |  |  |
| HBV DNA (copies/ml) | < 5x10^7^ | ***S****_hbvlow_* | *m_hbvlow_* | 11 | 2 | 0 | 7 | 2 | 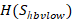 | 1.357 | 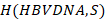 | 1.271 | 0.052 |
|  | ≥ 5x10^7^ | ***S****_hbvhigh_* | *m_hbvhigh_* | 7 | 1 | 0 | 5 | 1 | 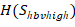 | 1.149 |  |  |  |
| PBMCs (cells/ml) | < 8x10^6^ | ***S****_pbmclow_* | *m_pbmclow_* | 11 | 2 | 0 | 7 | 2 | 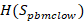 | 1.308 | 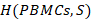 | 1.076 | 0.247 |
|  | ≥ 8x10^6^ | ***S****_pbmchigh_* | *m_pbmchigh_* | 6 | 0 | 1 | 5 | 0 | 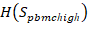 | 0.651 |  |  |  |

Multimedia Appendix 13**: The weighted sum of the entropy of the child nodes and the information gain reach its maximum value.** Cohen classified effect sizes on MTCT risk as trivial (d<0.2), small (0.2 ≤ d < 0.5), medium (0.5 ≤ d < 0.8), and large (d ≥ 0.8).
